# Sirtuins’ Deregulation in Bladder Cancer: SIRT7 Is Implicated in Tumor Progression through Epithelial to Mesenchymal Transition Promotion

**DOI:** 10.3390/cancers12051066

**Published:** 2020-04-25

**Authors:** Sara Monteiro-Reis, Ana Lameirinhas, Vera Miranda-Gonçalves, Diana Felizardo, Paula C. Dias, Jorge Oliveira, Inês Graça, Céline S. Gonçalves, Bruno M. Costa, Rui Henrique, Carmen Jerónimo

**Affiliations:** 1 Cancer Biology and Epigenetics Group, IPO Porto Research Center (CI-IPOP), Portuguese Oncology Institute of Porto (IPO Porto), Rua Dr. António Bernardino de Almeida, 4200-072 Porto, Portugal; sara.raquel.reis@ipoporto.min-saude.pt (S.M.-R.); ana.lameirinhas@ipoporto.min-saude.pt (A.L.); vera.miranda.goncalves@ipoporto.min-saude.pt (V.M.-G.); dianafelizardo@gmail.com (D.F.); paula.dias@ipoporto.min-saude.pt (P.C.D.); maria.ines.graca@ipoporto.min-saude.pt (I.G.); henrique@ipoporto.min-saude.pt (R.H.); 2 Master in Oncology, Institute of Biomedical Sciences Abel Salazar-University of Porto (ICBAS-UP), Rua de Jorge Viterbo Ferreira n.° 228, 4050-313 Porto, Portugal; 3 Department of Pathology, Portuguese Oncology Institute of Porto, 4200-072 Porto, Portugal; 4 Urologic Clinic, Portuguese Oncology Institute of Porto (IPO Porto), Rua Dr. António Bernardino de almeida, 4200-072 Porto, Portugal; jorge.oliveira@ipoporto.min-saude.pt; 5 Life and Health Sciences Research Institute (ICVS), School of Medicine, University of Minho, Campus de Gualtar, 4710-057 Braga, Portugal; celinegoncalves@med.uminho.pt (C.S.G.); bfmcosta@med.uminho.pt (B.M.C.); 6 ICVS/3B’s-PT Government Associate Laboratory, Braga/Guimarães, University of Minho, Campus de Gualtar, 4710-057 Braga, Portugal; 7 Department of Pathology and Molecular Immunology, Institute of Biomedical Sciences Abel Salazar-University of Porto (ICBAS-UP), Rua de Jorge Viterbo Ferreira n.° 228, 4050-313 Porto, Portugal

**Keywords:** bladder cancer, SIRT7, EMT

## Abstract

Sirtuins are emerging players in cancer biology and other age-related disorders, and their putative role in bladder cancer (BlCa) remains elusive. Further understanding of disease biology may allow for generation of more effective pathway-based biomarkers and targeted therapies. Herein, we aimed to illuminate the role of sirtuins’ family in BlCa and evaluate their potential as disease biomarkers and therapeutic targets. SIRT1-7 transcripts and protein levels were evaluated in a series of primary BlCa and normal bladder mucosa tissues. SIRT7 knockdown was performed through lentiviral transduction in MGHU3, 5637 and J82 cells and its functional role was assessed. SIRT1, 2, 4 and 5 expression levels were significantly lower in BlCa, whereas SIRT6 and 7 were overexpressed, and these results were corroborated by TCGA cohort analysis. SIRT7 transcript levels were significantly decreased in muscle-invasive vs. papillary BlCa. In vitro studies showed that SIRT7 downregulation promoted cells migration and invasion. Accordingly, increased EMT markers expression and decreased E-Cadherin (CDH1) was observed in those BlCa cells. Moreover, increased EZH2 expression and H3K27^me3^ deposition in E-Cadherin promoter was found in sh-SIRT7 cells. We demonstrated that sirtuins are globally deregulated in BlCa, and specifically SIRT7 downregulation is implicated in EMT, fostering BlCa invasiveness through EZH2-CDH1 axis.

## 1. Introduction

Bladder cancer (BlCa) is the 9th most common cancer type worldwide, with an estimated 400,000 new cases and 160,000 deaths per year, in both genders [1]. Men are more susceptible and in more developed regions, BlCa represents has the 6th highest incidence among different cancers. BlCa may be categorized according to clinical, pathological, or molecular characteristics. Muscle-invasive bladder cancer (MIBC) which accounts for about 20% of all cases, represents the more aggressive form, being more likely to progress and metastasize, whereas non-muscle-invasive bladder cancer (NMIBC) is mostly characterized by multiple local recurrences, which, over time, also entail increased risk of invasion. Indeed, although most newly diagnosed patients present NMIBC (approximately 80%), there is variable risk of progression, with increased morbidity [2,3,4].

Sirtuins (SIRTs) are a family of NAD^+^-dependent deacetylases highly conserved among all living organisms. Seven different SIRTs (SIRT1–7) are described in mammals, also known as Class III histone deacetylases (HDACs), which differing among each other in substrate specificity and catalytic activity [5]. Within the cell, these enzymes participate in control of important biological processes, including cell division, differentiation, metabolism, genomic stability, survival, senescence and organismal lifespan [6]. In addition, SIRTs expression is deregulated in many cancer types [7,8,9]. SIRT1 and SIRT3 may be up- or downregulated depending on the cancer type, acting either as oncogenes (e.g., colorectal or oral cancer) [10,11], or tumor suppressors (e.g., SIRT1 in bladder cancer, SIRT1 and SIRT3 in breast and prostate cancer) [12,13]. SIRT2 and SIRT4, on the other hand, are considered tumor suppressor, downregulated in glioma and hepatocellular carcinoma (SIRT2) [14,15], and bladder, gastric and lung cancer (SIRT4) [16], among others. Although little is known about the role of SIRT5 in neoplastic transformation, it is overexpressed in non-small-cell lung cancer (NSCLC) [17]. Concerning SIRT6, it is downregulated in several cancers, including hepatocellular carcinoma [18,19], but it is overexpressed in breast cancer and NSCLC [20,21]. Finally, an oncogenic function has been proposed for SIRT7 as it was found overexpressed in several epithelial cancers [22,23]. Moreover, SIRT7 is mostly localized in the nucleus and its deacetylase function needs to be disclosed, with only a few well characterized substrates reported [24,25]. SIRT7 deacetylase activity is related with histones, disclosing highly selective activity for lysine 18 of histone H3 (H3K18Ac), notwithstanding other protein targets involved in cell homeostasis and stress response [24]. SIRT7 is also involved in ribosomes biogenesis and other mechanisms of cell proliferation [26,27]. 

Although sirtuins have been characterized in various neoplasms, their putative role in BlCa development and progression remains elusive with only a few published studies to date [12,16,28]. Thus, we sought to comprehensively characterize SIRTs expression in BlCa tissues, comparing with normal bladder mucosa, assessing their potential as prognostic biomarkers. Furthermore, the phenotypic impact of SIRT7 deregulation in BlCa cells was also evaluated.

## 2. Results

### 2.1. Sirtuins Transcript Levels Characterization in Bladder Urothelial Carcinoma

**Figure 1:**
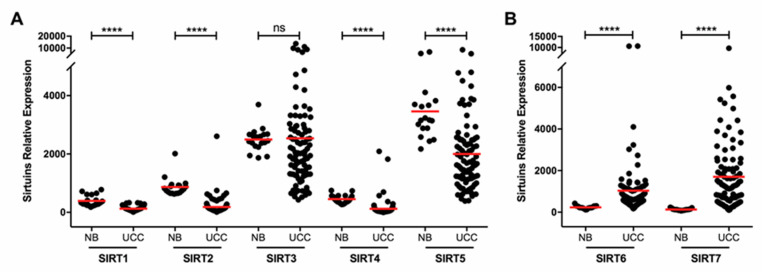
Sirtuin family transcript levels characterization in bladder urothelial carcinoma. Characterization of SIRT1, SIRT2, SIRT3, SIRT4 and SIRT5 (**A**), and SIRT6 and SIRT7 (**B**) in the bladder cancer and normal mucosae cohorts, by quantitative RT-PCR. **** *p* < 0.0001, ns—nonsignificant. UCC—urothelial cell carcinoma, NB—normal bladder mucosae.

Transcript levels of all sirtuins (SIRT1-7) were evaluated in 94 BlCa samples (UCC) by RT-qPCR and compared with normal mucosa (controls). Statistically significant differences were disclosed for all sirtuins, except for *SIRT3* (MW *p* = 0.0612; Figure 1A). Reduced *SIRT1*, 2, 4 and *5* expression levels were depicted in BlCa (MW *p* < 0.0001 for all; Figure 1A), whereas *SIRT6* and *SIRT7* were overexpressed (MW *p* < 0.0001 for both; Figure 1B). In TCGA dataset, SIRTs expression in BlCa compared to paired NB samples disclosed similar results, with a significant decrease of *SIRT1* and *SIRT3* expression (MW *p* < 0.0001 and *p* = 0.0422, respectively; Figure S1A), and significant increase in *SIRT6* and *SIRT7* expression in BlCa tissues (MW *p* < 0.0001 for both; Figure S1B).

### 2.2. SIRT7 Expression Is Decreased in Invasive and TCGA “Basal-Like” Urothelial Carcinoma

**Figure 2:**
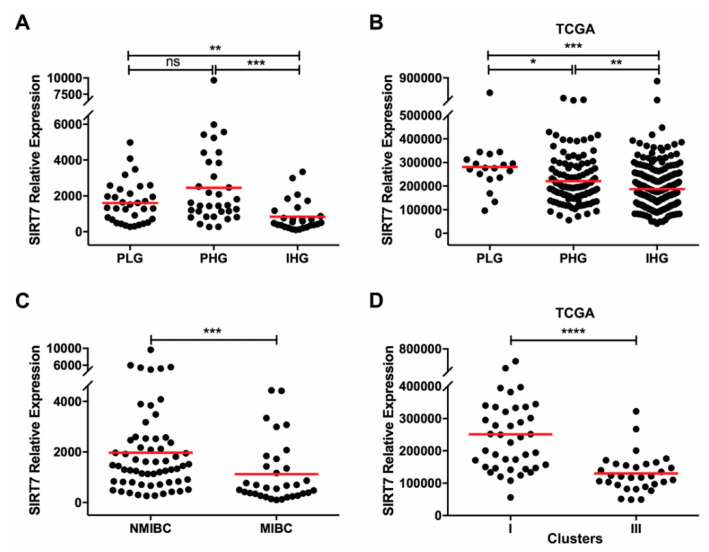

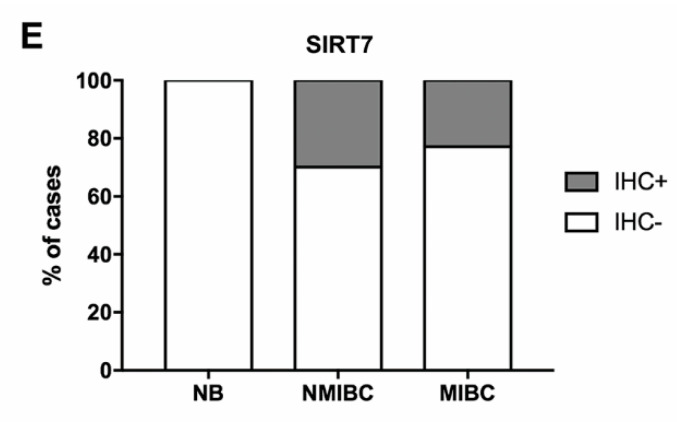
SIRT7 expression downregulation in invasive and TCGA “basal-like” urothelial tumors. Characterization of SIRT7 gene expression in the bladder cancer cohort (**A**) and TCGA cohort (**B**) categorized by clinical grade. Characterization of SIRT7 gene expression in the bladder cancer cohort categorized by non-muscle invasive and muscle invasive bladder cancer (**C**). SIRT7 gene expression according to TCGA molecular clusters analysis in the TCGA cohort (**D**). SIRT7 immunohistochemistry results for the normal and tumor tissue samples cohort, categorized by non-muscle invasive and muscle invasive bladder cancer, regarding the calculated immunoscore (**E**). * *p* < 0.05, ** *p* < 0.01, *** *p* < 0.001 and **** *p* < 0.0001. PLG—papillary low-grade, PHG—papillary high-grade, IHG—invasive high-grade, NMIBC—non-muscle invasive bladder cancer, MIBC-muscle invasive bladder cancer.

Characterization of SIRTs expression was then evaluated according to tumor subtype. Overall, lower transcript levels were observed in invasive high-grade carcinomas (IHG) comparing with papillary low-grade carcinomas (PLG) (Figure S2A), although statistical significance was only reached for *SIRT7* (KW *p* < 0.0001; Figure 2A). Additionally, significantly decreased *SIRT7* expression was also observed in IHG compared to papillary high-grade carcinomas (PHG) (Figure 2A). Contrarily, *SIRT4* expression levels were significantly higher in IHG compared to PLG (KW *p* = 0.0012; Figure S2A). The same analysis was also performed in a TCGA bladder urothelial cancer cohort and a similar SIRTs expression profile was found, with IHG showing significantly increased *SIRT4* expression levels comparing to PLG, whereas *SIRT5* and *SIRT6* expression levels were decreased (Figure S2B). Furthermore, in TCGA dataset, *SIRT7* expression was significantly lower in IHG compared to PHG and PLG (KW *p* < 0.0001 for both; Figure 2B), although no significant differences were disclosed between PLG and PHG.

Concerning pathological stage, two categories were considered: pTa-1/NMIBC (tumors confined to the bladder mucosa), and pT2-4/MIBC (tumors that invade the bladder muscular layer or beyond). In MIBC, *SIRT4* expression levels were significantly higher (MW *p* = 0.0009 s) and *SIRT7* levels were significantly lower (MW *p* = 0.0006; Figure 2C) comparing with NMIBC. In TCGA cohort, no statistically significant differences were disclosed, since only two cases are classified as NMIBC. Furthermore, in both IPO Porto’s and TGCA cohorts, no association was found between SIRTs expression levels and patients’ gender or age at diagnosis.

Since alterations in *SIRT7* altered expression were concordant in both cohorts, we further assessed the prognostic value of *SIRT7* expression. Of the 94 patients enrolled, four were lost to follow-up. The median follow-up time of BlCa patients was 72 months (range: 1–248 months). At the last follow-up time point, 44 patients were alive with no evidence of cancer, eight patients were alive with disease, 10 had died from other causes and 28 had deceased due to BlCa. In IPO Porto’s cohort, high tumor grade and pathological stage, as well as more advanced age at diagnosis, were significantly associated with shorter overall survival in multivariable Cox-regression model (*p* = 0.031, *p* = 0.037 and *p* = 0.030, respectively). Although *SIRT7* expression levels did not associate with patients’ prognosis in IPO Porto’s cohort, in TCGA dataset, cases with lower *SIRT7* expression (percentile 25) disclosed shorter overall survival, although only in univariable analysis (*p* = 0.028). Moreover, sirtuins’ expression did not associate with disease-free survival, both considering the total cohort of patients and in patients without (NMIBC) or with (MIBC) invasive disease, separately.

Furthermore, TCGA clusters for molecular markers signatures in BlCa were also carried out. These clusters categorize samples using various known molecular characteristics. Cluster I subset consists of tumors with “papillary-like” morphology and higher expression of epithelial markers like E-cadherin (ECAD), whereas cluster III is characterized by low ECAD expression and high cytokeratins expression, consistent with a “basal-like” phenotype [29]. *SIRT7* expression was significantly lower in “basal-like” phenotype (cluster III) than in “papillary-like” phenotype (cluster I) (MW *p* < 0.0001, Figure 2D).

Immunoexpression analysis showed that both normal urothelial and BlCa cells expressed nuclear SIRT7 (Figure S3). Although no significant correlation was found between *SIRT7* mRNA and protein levels, higher staining intensity and/or percentage of positive cells was observed in BlCa compared to normal urothelium (Figure 2E). Furthermore, a slight reduction of SIRT7 expression in MIBC was depicted (Figure 2E), paralleling *SIRT7* transcript level results.

### 2.3. SIRT7 Expression in Bladder Cancer Cell Lines

**Figure 3:**
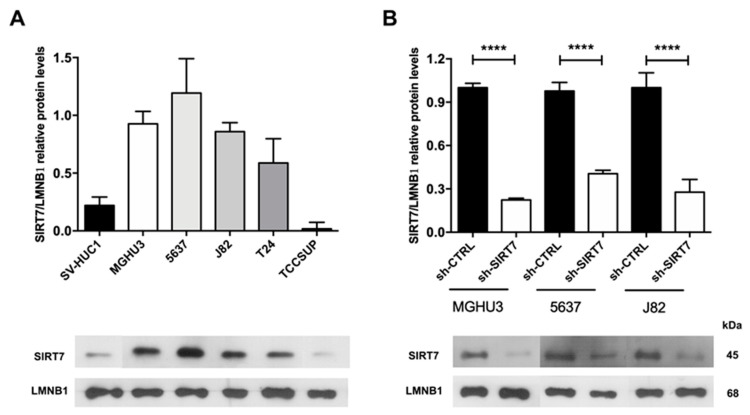
SIRT7 expression in bladder cancer cell lines. Expression of SIRT7 nuclear protein (**A**) in bladder cancer cell lines by Western blot; results are representative of three independent experiments with mean ± SD. Confirmation of SIRT7 knockdown in MGHU3, 5637 and J82 cell lines at nuclear protein level (**B**) by Western blot; **** *p* < 0.0001, results are representative of three independent experiments with mean ± SD.

SIRT7 nuclear protein levels were evaluated in five BlCa cell lines and one immortalized normal urothelial cell line (SV-HUC1), where MGHU3, J82 and 5637 cells displayed the highest SIRT7 protein levels (Figure 3A). The lowest levels were found in the more aggressive cell line, namely TCCSUP cell line derived from a Grade IV carcinoma, whereas MGHU3 derived from a Grade I carcinoma, 5637 from a Grade II carcinoma, and J82 cell line originated from a Grade III carcinoma. 

Because these three cell lines disclosed the highest SIRT7 nuclear protein expression, they were chosen for lentiviral downregulation experiments. Before transfection, SIRT7 nuclear localization was confirmed by immunofluorescence for the three selected cell lines (Figure S4). Furthermore, after lentiviral transfection, a significant reduction was achieved for the three cell lines (MW *p* < 0.0001; Figure 3B), and reduced SIRT7 nuclear expression was confirmed by immunofluorescence in sh-SIRT7 cells compared to sh-scramble/CTRL cells s.

### 2.4. SIRT7 Downregulation Promotes Invasiveness and EMT in Bladder Cancer Cells

**Figure 4:**
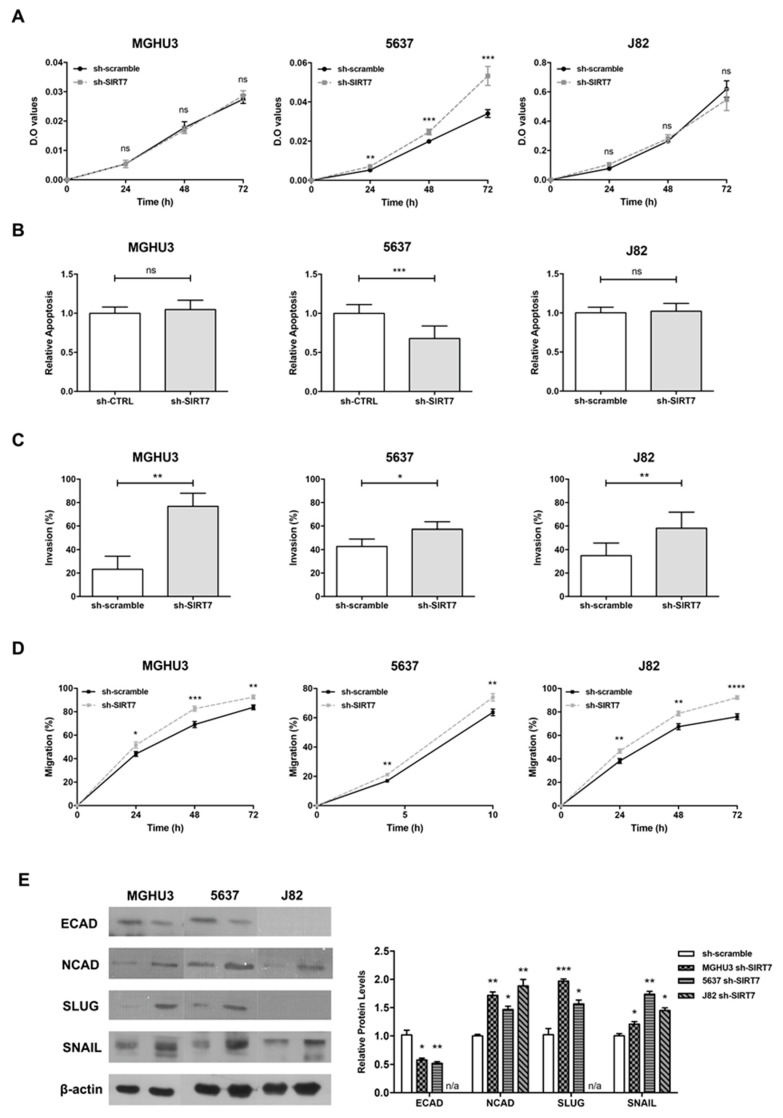
SIRT7 downregulation promotes invasiveness and EMT in bladder cancer cells. Effect of SIRT7 knockdown for MGHU3, 5637 and J82 cell lines at (**A**) cell viability by MTT assay, (**B**) apoptosis- cell death by APOPercentage assay, (**C**) cell invasion by BD Biocoat Matrigel Invasion Chambers and (**D**) cell migration by wound-healing assay; * *p* < 0.05, ** *p* < 0.01, *** *p* < 0.001 and **** *p* < 0.001; results are representative of three independent experiments with mean ± SD, each of them in triplicates. Expression of epithelial and mesenchymal markers and EMT transcription factors (**E**) in MGHU3, 5637 and J82 SIRT7 knockdown by western blot; results are representative of three independent experiments with mean ± SD.

Although no significant alterations in cell proliferation (Figure 4A) and apoptosis (Figure 4B) were found in sh-SIRT7 vs. sh-scramble/CTRL MGHU3 and J82 cells, 5637 sh-SIRT7 displayed a higher proliferation rate (especially at the 48 h time-point), and reduced apoptosis levels (*p* < 0.001 and *p* < 0.01, respectively). Moreover, a significant increase in cell migration was observed at all time points in MGHU3, 5637 and J82 sh-SIRT7 cells (Figure 4C), and the same was depicted for cell invasion (Figure 4D).

Moreover, sh-SIRT7 cells disclosed E-cadherin (or ECAD, an epithelial marker) decreased expression compared to wild type cell lines that expressed this protein (MGHU3 and 5637), whereas significantly increased N-cadherin (or NCAD, mesenchymal marker) protein levels were found in all tested cell lines. Moreover, these results were corroborated by immunofluorescence analysis for the same markers in the same cell lines (Figure S5). Furthermore, EMT transcription factors, SLUG and SNAIL, paralleled the same expression pattern as ECAD in the same cell lines (Figure 4E).

### 2.5. SIRT7 Downregulation Associates with E-Cadherin Repression Mediated by Histone Methyltransferase EZH2

Because a global increase in both invasion and migration were found in sh-SIRT7 cell lines, with a concomitant decrease of the epithelial marker and key EMT player ECAD (*CDH1* gene), we further investigated the expression of *CDH1* in tissue samples from IPO Porto’s cohort. Indeed, MIBC showed decreased *CDH1* transcript levels and *CDH2* upregulation (MW *p* < 0.0001 and *p* = 0.0011, respectively; Figure S6). Moreover, *SIRT7* and *CDH1* transcript levels positively correlated (r= 0.58, 95% CI 0.422 to 0.704, *p* < 0.0001) whereas *SIRT7* and *CDH2* transcript levels negatively correlated (r= −0.22, 95% CI −0.403 to −0.00187, *p* < 0.05) in MIBC patients.

**Figure 5:**
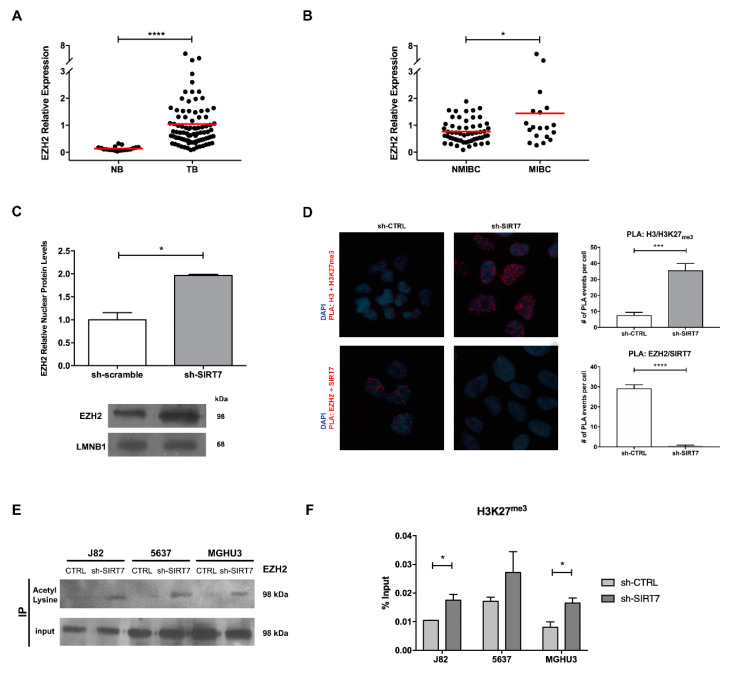
SIRT7 downregulation associates with E-Cadherin repression mediated by histone methyltransferase EZH2. Characterization of EZH2 gene expression in the bladder cancer and normal mucosae cohort (**A**), and in non-muscle invasive and muscle-invasive bladder cancer cases (**B**), by quantitative RT-qPCR. Characterization of EZH2 protein expression (**C**) in 5637 sh-CTRL and sh-SIRT7 cells, by western blot analysis. Proximity Ligation Assay for assessment of interaction of Histone 3 with Histone 3 lysine 27 tri-methylation (H3K27^me3^) and EZH2 with SIRT7, in 5637 sh-scramble and sh-SIRT7 cells (100× magnification) (**D**). Western blot analysis for EZH2 protein, after co-immunoprecipitation assay with acetyl-lysine antibody in J82, 5637 and MGHU3 sh-scramble/CTRL and sh-SIRT7 cells (**E**). Chromatin immunoprecipitation results for H3K27me3 deposition across the CDH1 gene promoter, in MGHU3, 5637 and J82 sh-scramble/CTRL and sh-SIRT7 cells (**F**). * *p* < 0.05, *** *p* < 0.001 and **** *p* < 0.0001.

As *CDH1*, which is transcriptionally regulated by *EZH2* [a SIRT7 substrate [30]], was found decreased in MIBC cases, and taking into account the previous results in SIRT7 modulated cell lines (Figure 4), we decided to explore the interplay between SIRT7, EZH2 and CDH1/ECAD. Indeed, *EZH2* transcript levels were significantly higher in BlCa tissues compared to NB samples (MW *p* < 0.0001, Figure 5A). Furthermore, MIBC depicted the highest *EZH2* transcript levels (MW *p* = 0.0444, Figure 5B), and an inverse expression pattern was depicted for SIRT7 and EZH2 transcripts in MIBC (Figure S7). Moreover, EZH2 protein levels were significantly increased in sh-SIRT7 5637 cells (chosen because it showed differences in all phenotypic assays), compared to sh-CTRL cells (MW *p*< 0.01, Figure 5C). 

Additionally, since EZH2 represses several genes, including CDH1, through its histone methyltransferase activity, especially by histone 3 lysine 27 tri-methylation (H3K27^me3^) deposition within the respective promoters, PLA, co-immunoprecipitation (co-IP) and ChIP assays were performed. Firstly, PLA assay showed that EZH2 and SIRT7 physically interact in sh-CTRL 5637 cells (*p* < 0.0001; Figure 5D), and that sh-SIRT7 cells showed more H3K27^me3^ mark (*p* < 0.001; Figure 5D). Next, a co-IP with an acetylated-lysine antibody disclosed higher acetylated EZH2 in sh-SIRT7 cells, comparatively to sh-CTRL cells (Figure 5E). Lastly, a ChIP assay was performed to assess the deposition of H3K27^me3^ mark at CDH1 promoter region in all transfected cell lines. As expected, increased H3K27^me3^ was observed across the CDH1 promoter in sh-SIRT7 cells, with a significant increase in both MGHU3 and J82 cells (2-way ANOVA *p* = 0.01; Figure 5F), suggesting that CDH1 repression associated with SIRT7 downregulation occurs through EZH2 overexpression.

## 3. Discussion

Sirtuins, also known as Class III HDACs, are involved in many biological processes, including cell division, differentiation, metabolism, genomic stability, survival, senescence and organismal lifespan [6], and variable SIRTs deregulated expression has been reported in many cancer types [7,8,9]. Remarkably, sirtuins may act either as oncogenes or tumor suppressor genes in different tumor models [12,13,14,15,16]. Thus, better understanding of the biological role of these unique enzymes in tumorigenesis might provide novel biomarkers for disease management as well as putative therapeutic targets.

Herein, we report, for the first time, a comprehensive evaluation of sirtuins (*SIRT1-7*) mRNA expression in a series of 94 BlCa cases from a single institution and respective validation in TCGA dataset, comparing with normal bladder mucosa. Significant differences were depicted for all sirtuins, except for *SIRT3*, with *SIRT1*, *2*, *4* and *5* downregulation and *SIRT6* and *7* overexpression. These findings were mostly validated in TCGA dataset, especially for *SIRT6* and *SIRT7*. Previous studies on BlCa have mainly focused on SIRT1 and SIRT4 and were mostly based in publicly available datasets only [12,16], not providing a global picture of sirtuin deregulation in BlCa. Interestingly, besides significant differences between BlCa and urothelium, differential expression of some sirtuins was also disclosed between tumors with dissimilar clinical and biological behavior. Interestingly, although SIRT7 was overexpressed in BlCa, the more aggressive tumors (IHG) disclosed significantly lower expression levels compared to PLG and PHG, both in IPO Porto’s and TCGA cohorts. Furthermore, in MIBC both *SIRT7* transcript and protein disclosed a significant reduction compared to NMIBC. Remarkably, previous reports on SIRT7 in uterus, colon, kidney, ovary and prostate cancers revealed increased expression levels [22,24]. Nevertheless, in all those models, a strict oncogenic role was proposed for *SIRT7*, whereas our findings suggest that, at the least in bladder carcinogenesis, *SIRT7* may play a dual role, eventually context-dependent. Furthermore, although we did not find significant differences in *SIRT6* transcript levels between PHG and IHG tumors (either in our and TCGA cohort), nor between different stages of MIBC, Wu et al. reported that SIRT6 protein levels declined significantly from T2 to T4 MIBC, which also suggests that the functional importance of sirtuins may change along cancer progression [28]. The observed decreased overall survival in BlCa patients with lower *SIRT7* expression in TCGA cohort (eventually associated with higher grade and stage, as well as molecular BlCa subtype) further suggests that decreased SIRT7 impacts on neoplastic cell biology, promoting a more aggressive phenotype.

Taking in consideration *SIRT7* expression patterns in normal and neoplastic urothelium, we sought to characterize the effects of its deregulated expression at molecular level. Thus, after characterization of SIRT7 transcript and protein expression levels in neoplastic and benign urothelial cell lines, three cell lines were chosen for further experiments as their profile more closely replicated that of a spectrum of BlCa tissues. Interestingly, in vitro phenotypic assays demonstrated that although SIRT7 downregulation did not affect cell proliferation or apoptosis, with the exception of 5637 cell line, rather impairing cell motility, decreasing both cell migration and cell invasion capabilities in all modulated cell lines. These effects immediately suggested a putative association between SIRT7 and EMT, a process that is key for tumor invasion and metastization [31,32]. This hypothesis was confirmed as SIRT7 knockdown significantly associated with decreased E-Cadherin expression and augmented expression of a mesenchymal marker (N-Cadherin), and EMT-inducing transcription factors (SLUG and SNAIL), in the modulated BlCa cells. Although only a few studies investigated the relationship between sirtuins and EMT [33], SIRT7 depletion in PC3 prostate cancer cell line was shown to impair migration and invasiveness, reprograming neoplastic cells towards epithelial gene expression [22]. Our results indicate the opposite trend in BlCa cells, which might be due to the pleiotropic effects of sirtuins and/or the dissimilar molecular profile of prostate and urothelial cancer cells [34]. 

Remarkably, we found that the mechanism by which SIRT7 affects CDH1 expression, and thus EMT, is probably linked to EZH2. EZH2 is a well-known member of the polycomb repressive complex 2 (PRC2), described as being involved in the transcription repression by catalyzing the repressive H3K27^me3^ mark at several gene promotes, including *CDH1* [35,36,37]. Previously, proteomic analyses demonstrated that among 250 candidate substrates, EZH2 was a SIRT7 substrate [30,38]. In our study, sh-SIRT7 cells showed increased total and acetylated EZH2 expression, followed by decreased ECAD protein. Concurrently, increased H3K27^me3^ deposition at CDH1 promoter was also observed in the same cells. Thus, when SIRT7 is downregulated, EZH2 activity might be enhanced by acetylation, contributing to *CDH1* transcription repression through H3K27^me3^ deposition in its promoter, as previously reported [30]. *CDH1* repression and concomitant EMT transcription factors’ upregulation (e.g., SNAIL and SLUG), might then lead to a shift from epithelial to mesenchymal phenotype, allowing for increased cancer cell motility. Indeed, upregulation of these specific EMT transcription factors, due to diverse upstream signals and post-transcriptional mechanisms, also corroborates our hypothetic mechanism. Indeed, both SNAIL and SLUG were shown to cooperate with PRC2, and specifically with EZH2, towards controlling the expression of several genes, relevant for neural crest development, including CDH1 [39,40]. Moreover, during EMT, Snail was proven to recruit EZH2 to specific genomic sites by the enrollment of the long non-coding RNA HOTAIR [41]. Thus, our results suggest that EMT transcription factors’ upregulation in sh-SIRT7 BlCa cells might be due to the phenotypic shift in invasion and migration and, at the molecular level, by recruitment of EZH2 to specific targets.

Thus far, only a limited number of upstream SIRT7 transcription regulators, such as histone deacetylase 3 (HDAC3) and the X-box binding protein 1 (XBP1) molecules have been identified [42,43]. At post-transcriptional level, SIRT7 was shown be negatively regulated by microRNAs, such as those from microRNA-125 family [44]. However, few reports deal with SIRT7 regulation by post-translational modifications [45,46,47]. Hence, it would be important to further explore how regulation of SIRT7 occurs in BlCa, and unveil how SIRT7 expression shift occurs from non-invasive to invasive BlCa.

Moreover, although discovery of new prognostic biomarkers for BlCa is imperative for more effective disease management, the aim of our study was mostly to uncover how expression of all sirtuins was altered in BlCa, and to investigate whether they might be implicated in bladder carcinogenesis and/or disease progression and invasiveness. Indeed, we were able to demonstrate that for SIRT7. Nonetheless, the analyzed cohort was composed by patients diagnosed over a large time span (1991–2011) and the small number of events occurring in this cohort precluded a more robust and detailed statistical analysis. 

Overall, our results suggest that increased *SIRT7* expression occurs during urothelium neoplastic transformation, which usually results in the formation of non-invasive, papillary neoplasms or flat lesions like urothelial carcinoma in situ [48]. At this stage, it is likely that SIRT7 is involved in promoting cell growth and survival, which are key to neoplastic development, eventually through deacetylation of H3K18 [24]. Then, transition to an invasive phenotype might require *SIRT7* downregulation, involving EZH2 upregulation and acetylation, among other mechanisms, which promote EMT. Although the mechanism of SIRT7 deregulation in BlCa remains elusive, it is tempting to speculate whether it might be due to epigenetic mechanisms, which allow for the suggested plasticity of *SIRT7* gene expression during carcinogenesis and tumor progression.

## 4. Materials and Methods 

### 4.1. Patients and Samples

Patients with primary bladder urothelial carcinoma (UCC), treated with transurethral resection (TUR) or radical cystectomy, between 1991 and 2011 at Portuguese Oncology Institute of Porto (IPO Porto), Portugal (*n* = 94). A set of 19 morphologically normal bladder mucosa (NB) tissue samples was obtained from BlCa-free individuals (prostate cancer patients submitted to radical prostatectomy) and served as controls. All specimens were fresh-frozen at −80 °C and subsequently cut in a cryostat for confirmation of representativity and nucleic acid extraction. From each specimen, fragments were collected, formalin-fixed, and paraffin embedded for routine histopathological examination, including grading and pathological staging, by a dedicated uropathologist [49]. Relevant clinical data was collected from clinical charts (Table 1). Patients and controls were enrolled after informed consent. This study was approved by the institutional review board (Comissão de Ética para a Saúde) of IPO Porto (CES103-14).

### 4.2. Real-Time Quantitative PCR (RT-qPCR)

RNA was extracted from tissues and from MGHU3, 5637 and J82 sh-scramble and sh-SIRT7 cells using TRIzol^®^ (Invitrogen, Carlsbad, CA, USA), according to manufacturer’s instructions. For tissue RNA, cDNA synthesis was performed using the High Capacity cDNA Reverse Transcription Kit (Applied Biosystems^®^, Foster City, CA, USA), according to manufacturer’s instructions. Sirtuins transcript levels were quantified by RT-qPCR. Expression levels were evaluated using 4.5 µL of diluted cDNA, 5 µL of TaqMan^®^ Universal PCR Master Mix No AmpErase^®^ UNG (Applied Biosystems^®^) and 0.5 µL of TaqMan^®^ Gene Expression Assay, specific for each sirtuin and reference genes, as described in Table S1 (Applied Biosystems^®^). Each sample was run in triplicate and the RT-qPCR conditions were: 2 min at 50 °C, followed by enzyme activation for 10 min at 95 °C, and 45 cycles which included a denaturation stage at 95 °C for 15 s and an extending stage at 60 °C for 60 s onds. *HPRT* and *SDHA* were both used as reference genes for normalization. Relative expression of target genes tested in each sample was determined as: [Gene Expression Level = (Gene Mean Quantity/(*HPRT1* & *SDHA*) Mean Quantity) × 1000].

Concerning cell lines, 1000 ng of RNA were reverse transcribed using RevertAid RT kit (Thermo Fisher Scientific Inc., Waltham, MA, USA), according to manufacturer’s instructions. For 100 ng of cDNA, *SIRT7* and *NCAD* transcript levels were quantified using TaqMan^®^ Gene Expression Assay, as described above, in 396 well plates LightCycler480II (Roche, Basel, Switzerland). For *ECAD* and *EZH2* genes, transcription levels were also evaluated in J82 sh-scramble and sh-SIRT7 cells in 396 well plates LightCycler480II (Roche) using Xpert Fast SYBER Mastermix Blue (GRiSP Research Solutions, Porto, Portugal) with specific primers (S2). Transcript levels for studied genes were then evaluated using ΔΔCt method, with *HPRT* and *BGUS* housekeeping genes as reference genes.

### 4.3. Immunohistochemistry

Immunohistochemistry was performed using the Novolink™ Max Polymer Detection System (Leica Biosystems, Wetzlar, Germany]. Three-μm thick tissues s tions from formalin-fixed and paraffin-embedded BlCa (corresponding to 88 of the 94 cases, for which there was archived tissue available) and controls (*n* = 25, consisting of normal urothelial mucosa collected from the urether of nephrectomy specimens with renal cell tumors) were cut, deparaffinized and rehydrated. Antigen retrieval was accomplished by microwaving the specimens at 800 W for 20 min in 10 mM sodium citrate buffer, pH = 6. Endogenous peroxidase activity was blocked by incubating the s tions in 0.6% hydrogen peroxide solution for 20 minutes. Primary monoclonal antibody for *SIRT7* (HPA053669, Sigma-Aldrich™, St. Louis, MO, USA) was used in 1:500 dilution, and incubated at room-temperature (RT) for one hour. Then, 3,3′-diaminobenzidine (Sigma-Aldrich™) was used as chromogen for visualization and slides were mounted with Entellan^®^ (Merck-Millipore, Burlington, MA, USA). Normal testicular tissue, showing intense SIRT7 immunoreactivity was used as positive control. SIRT7 immunoexpression was evaluated by a dedicated uropathologist and cases were classified using a semi-quantitative scale for both staining intensity (0—no staining; 1—intensity lower than normal urothelium; 2—intensity equal to normal urothelium; 3—intensity higher than normal urothelium) and percentage of positive cells (0—< 10%; 1—10–33%; 2—33–67%; 3— > 67%), in each tumor. Staining intensity and percentage of positive cell results were then combined in a single score (Score S = staining intensity x percentage of positive cells) assigned to each tumor, and further stratified into low expression (S < 4 = IHC−) and high expression (S ≥ 4 = IHC+ ) groups, which correspond to cases with less than 33% stained cells or staining intensity lower than normal urothelium, and cases with at least 33% stained cells with an intensity equal to or higher than normal urothelium.

### 4.4. TCGA Dataset Analysis in Bladder Urothelial Carcinoma Patients

The Cancer Genome Atlas (TCGA) dataset was interrogated for data on *SIRT1-7* expression and clinical information, when available, of 408 BlCa patients and 19 matched controls. All expression data from samples hybridized at the University of North Carolina, Lineberger Comprehensive Cancer Center, using Illumina HiSeq 2000 RNA Sequencing version 2 analysis, were downloaded from the GDC data portal (https://portal.gdc.cancer.gov/). Biospecimen Core Resources (BCRs) provided the clinical data of each patient. This data is available for download through the GDC data portal (https://portal.gdc.cancer.gov) (Table 2).

### 4.5. Cell Lines Culture

5637, J82, T24 and TCCSUP BlCa cell lines and normal bladder cell line SV-HUC1 were selected for this study. All cell lines were purchased from ATCC and grown using recommended medium (Biochrom-Merck, Berlin, Germany) supplemented with 10% fetal bovine serum (FBS, Biochrom) and 1% penicillin/streptomycin (GIBCO, Invitrogen) at 37 °C and 5% CO_2_. Mycoplasma test was regularly performed for all cell lines using TaKaRa PCR Mycoplasma Detection Set (Clontech Laboratories, Mountain View, CA, USA).

### 4.6. Lentiviral Transduction

*SIRT7* knockdown was performed through lentiviral transduction in J82 cell line using GIPZ^TM^ Lentiviral shRNA particles (Dharmacon^TM^, Lafayette, CO, USA), and in MGHU3 and 5637 cell lines using SMARTvector^TM^ Inducible Lentiviral shRNA particles (target sequence: 5’-CCCTGCGTGCTGGTGAAGA-3’). All sh-SIRT7 vectors included the green fluorescent protein (GFP). Briefly, cells were seeded in 12 well/plate at density of 4 × 10^4^ cells/well and incubated during 24 h in humidified chamber at 37 °C and 5% CO_2_. Then, culture medium was removed and 500 µL of completed medium with 8 µg/mL polybrene and lentiviral sh-SIRT7 particles with MOI 10 concentration were added. After 48 h, 1 µg/mL of puromycin dihydrochloride (Clontech Laboratories) was added to select stably transfected cells. For MGHU3 and 5637 cells, after puromycin selection, a treatment was performed with 100 ng/mL doxycycline in order to induce the Tet-On 3 G bipartite induction system. Additionally, J82, MGHU3 and 5637 control cells were generated using a non-target scramble shRNA under the same previously described conditions.

For clone selection, 10, 20 and 50 cells/well were seeded in 96 well plate after stable selection, and the isolated clones were grown until confluence for protein extraction, and subsequent western blot analysis for SIRT7 expression. Moreover, sh-SIRT7 cells were observed under the fluorescence microscope for GFP expression.

### 4.7. Protein Extraction

BlCa cell lines, sh-scramble/CTRL and sh-SIRT7 cells were grown until 80% confluence and homogenized in Kinexus lysis buffer supplemented with proteases inhibitors cocktail. Then, cells were sonicated for 5 cycles of 30 s ON and 30 s OFF (Bioruptor^®^, Diagenode, Liège, Belgium). After centrifugation, the supernatant was collected, and total protein was quantified according the Pierce BCA Protein Assay Kit (Thermo Fisher Scientific Inc.), according to the manufacture procedure. 

For subcellular fractionation, Nuclear Extract kit (Active Motif, Carlsbad, CA, USA) was used. Briefly, bladder cancer cell lines, sh-scramble/CTRL and sh-SIRT7 cells were washed in 1 X PBS with phosphate inhibitors and scrapped. Subsequently, cells were suspended in hypotonic buffer and incubated on ice during 15 min. Additionally, a detergent was added, and samples were centrifuged at 14,000 rpm during 30 s at 4 °C. Supernatant (cytoplasmic fraction) was collected and stored at −80 °C until use. Then, cell pellets were resuspended and incubated in a complete lysis buffer solution (lysis buffer with proteases inhibitor cocktail and 10 mM DTT), following centrifugation and supernatant (nuclear fraction) collection and storage at −80 °C. Nuclear and cytoplasmic proteins were then quantified using the Pierce BCA Protein Assay Kit (Thermo Fisher Scientific Inc.), according to manufacture procedure. 

### 4.8. Western Blot and Co-Immunoprecipitation

Aliquots of 30 µg total protein was separated in 10% polyacrylamide gel by SDS-PAGE and transferred onto an immunoblot PVDF membrane (Bio-Rad Laboratories, Hercules, CA, USA) in a 25 mM Tris-base/glycine buffer using a Trans-Blot Turbo Transfer system (Bio-Rad Laboratories). Membranes were blocked with 5% milk in TBS/0.1% Tween (TBS/T pH = 7.6) for 1 hour at RT. After incubation with primary antibodies for SIRT7 (1:350, HPA053669, Sigma-Aldrich) or EZH2 (1:500, NCL-L-EZH2, Leica Biosystems) for 1 h 30 min at RT, the membranes were washed in TBS/T and incubated with s ondary antibody coupled with horseradish peroxidase for 1 h at RT. The bound was visualized by chemiluminescence (Clarity WB ECL substrate, Bio-Rad) and quantification was performed using band densitometry analysis from the ImageJ software (version 1.6.1, National Institutes of Health). β-Actin (1:10,000, A1978, Sigma-Aldrich) for total protein and cytoplasmic protein analysis, and Laminin B1 (1:1000, D4Q42, Cell Signaling Technologies, Danvers, MA, USA) for nuclear protein, were used as loading controls. For co-immunoprecipitation assays, 200µg of total protein from cell lysates were incubated with anti-acetylated-lysine antibody (#9441, Cell Signaling Technology) and immunoprecipitated with Protein A/G magnetic beads (#16-663, Sigma-Aldrich) overnight at 4 °C. The final eluates were blotted with EZH2 primary antibody, as detailed above. Detailed information about western blot can be found at Figure S8.

### 4.9. Immunofluorescence (IF)

Wild-type MGHU3, 5637 and J82, sh-scramble/CTRL and/or sh-SIRT7 cells were seeded on cover slips at 20,000 cells/well, overnight. Briefly, cells were fixed in methanol during 10 min and then blocked with 5% bovine serum albumin (BSA) during 30 min. After overnight SIRT7 (1:500, HPA053669, Sigma-Aldrich), ECAD (1:150, #3195, Cell Signaling Technology) and/or NCAD (1:50, #13116, Cell Signaling Technology) incubation at RT, cells were incubated with s ondary antibody anti-rabbit IgG-TRITC (1:500, T6778, Sigma-Aldrich) during 1 h at RT. Finally, after 1× PBS wash, cells were stained with 4’,6-diamidino-2-phenylindole (DAPI) (AR1176, BOSTER Biological Technologies (Pleasanton, CA, USA) r in mounting medium. Pictures were taken on a IX51 fluorescence microscope (Olympus, Tokyo, Japan) equipped with an Olympus XM10digital camera using CellSens software.

### 4.10. Chromatin Immunoprecipitation (ChIP)

Chromatin immunoprecipitation (ChIP) analysis was performed in sh-scramble/CTRL and sh-SIRT7 cells. For the crosslink step, formaldehyde solution (Sigma) was added to adherent cells (~1 × 10^7^) media at 1% final concentration, and after an 8 minutes’ incubation at RT, reaction was immediately stopped by adding 1.5 mL of 2.5 M glycine and incubating for 5 min. Cells were then washed twice with ice-cold 1× PBS, scraped, harvested and centrifuged at 4 °C. 

Cell pellets were homogenized with cell lysis buffer (10 mM Tris-HCL pH7.5, 10 mM NaCL, 0.5% NP-40) and left on ice for 1 h30, with intermittent vortexing, and then centrifuged at 4 °C. At this point, pellets were re-suspended in nuclei lysis buffer (50 mM Tris-HCL pH = 7.5, 10 mM EDTA pH = 8, 1% SDS) and incubated for 15 min on ice, followed by adding of 2× volumes of IP dilution buffer (16.7 mM Tris-HCL pH7.5, 167 mM NaCl, 1.2 mM EDTA pH = 8, 0.01% SDS). Chromatin was solubilized and sheared to 200–400 bp fragments using an ultra-sonicator (Bioruptor^®^, Diagenode) for 15 cycles of 30 s ON and 30 s OFF. Soluble chromatin was then centrifuged and stored at −80 °C until further use. 

Before immunoprecipitation (IP), each 50 µL of chromatin was 1:10 diluted in dilution buffer (1.2 mM EDTA pH = 8, 16.7 mM Tris pH = 8, 167 mM NaCl, 1.1% Triton X-100, 0.01% SDS), and 5 µL of this solution was reserved in a new tube for the input control. After this, 20 µL of protein A+G magnetic beads (Millipore) were added to each IP sample, as well as ChIP-grade antibodies for Histone H3 (ab1791, Abcam, Cambridge, UK), tri-methylation of lysine 27 of histone H3 (H3K27me^3^, 07-449, Millipore), positive control (RNA polymerase II) and negative control (mouse IgG), at assay dependent concentration. IPs were incubated overnight at 4 °C with rotation. After incubation, magnetic beads were precipitated using 1.5 mL tubes magnet rack and washed with four different salt concentration buffers. At this point, elution buffer (50 mM Tris-HCL pH = 7.5, 10 mM EDTA pH = 8, 1% SDS) was added to all samples and input control, as well as 200 µg/mL of RNAse A, following an incubation of 30 min at 37 °C. After this, samples were incubated with proteinase K for 2 h at 62 °C, followed by an incubation of 10 min at 95 °C, for cross-link reversion. 

DNA was extracted from samples using the Qiaquick gel extraction kit (Qiagen, Hilden, Germany), according to manufacture procedures, and stored at −20 °C until further use. For qPCR, two pairs of primers for CDH1 promoter were designed, both for ~325 bp before TSS (F—5′-TAACCCACCTAGACCCTAGCAA-3′, R–5′-GCTGATTGGCTGAGGGTTCA-3′) and for ~600 bp before TSS (F—5′-ACCTGTACTCCCAGCTACTAGA-3′, R—5′-GATGGGGTCTCACTCTTTCACC-3′). RT-qPCR was performed as mentioned above, and the relative amount of promoter DNA was normalized using Input Percent method.

### 4.11. Proximity Ligation Assay (PLA)

Sh-scramble/CTRL and sh-SIRT7 cells, were seeded in 1 cm^2^ coverslips and allowed to grow overnight. Then, cells were fixed in 4% formaldehyde (Sigma) for 10 min and permeabilized in 0.5% Triton X-100 (Sigma), for 5 min, at RT and gently stirred. PLA assay was performed using the commercial kit Duolink In Situ (OLINK Bioscience, Uppsala, Sweden), according to manufacturer’s instructions. The antibodies used were Histone H3 (ab1791, Abcam, Cambridge, UK), tri-methylation of Lysine 27 of Histone H3 (H3K27me^3^, 07-449, Millipore), SIRT7 (HPA053669, Sigma-Aldrich) and EZH2 (NCL-L-EZH2, Leica Biosystems). After the procedure, cells were stained with 4′,6-diamidino-2-phenylindole (DAPI) (AR1176, BOSTER Biological Technology, Pleasanton, CA, USA) in mounting medium. Pictures were taken on an Olympus IX51 fluorescence microscope equipped with an Olympus XM10 digital camera using CellSens software. 

### 4.12. Cell Viability Assay

To assess the role of SIRT7 in cell growth, 3-(4,5-dimethylthiazol-2-yl)-2,5-diphenultetrazolium (MTT) assay (Sigma-Aldrich) was performed. Briefly, sh-scramble/CTRL and sh-SIRT7 cells were seeded at 3000 cell/well density, overnight, in 96 well plate. Then, 5 µg/mL MTT solution in completed MEM medium was incubated during 1 h at 37 °C for 0 h, 24 h, 48 h and 72 h. Then, formazan crystals formed were dissolved in dimethyl sulfoxide (DMSO) and spectrophotometric measurement was done at 540 nm, using 655 nm as a reference absorbance (Fluostar Omega, BMG Labtech, Offenburg, Germany). The optical density (OD) obtained for 24 h, 48 h and 72 h was normalized for the 0 h time point. At least three independent experiments were performed.

### 4.13. Apoptosis Assay

Apoptosis was assessed using the APOPercentage^TM^ kit (Biocolor Ltd., Belfast, Northern Ireland, UK). This assay uses a dye that is integrated by cells undergoing at early stage of apoptosis due to phosphatidylserine transmembrane movements, which results in APOPercentage dye incorporation by cells. Briefly, sh-scramble/CTRL and sh-SIRT7 cells were seeded in 24 well plate at density of 25,000 cell/well and incubated during 72 h in a humidified chamber at 37 °C and 5% CO_2_. At this time point, cells were incubated with 300 µL/well of APOPercentage dye solution at ratio 1:20 respectively, during 20 min at 37 °C. Then, cells were washed with PBS1 X and detached from well plate with Tryple^TM^ Express (GBICO) during 10 min at 37 °C. After that, APOPercentage Dye Release reagent was added and plate were vigorously agitated during 15 min, following colorimetric measurement at 550 nm with 620 nm reference filter (Fluostar Omega). The H_2_O_2_ was used as a positive control. The OD obtained for apoptosis assay was normalized for the OD obtained by viability assay at the same time point. At least three independent experiments were performed. 

### 4.14. Wound Healing Assay

Cells were seeded in 6 well plate at a density of 7.5 × 10^5^ cell/well and allowed reach confluence at 37 °C, 5% CO_2_. Then, a “wound” was made by manual scratching with a 200 µL pipette tip and cells were gently washed with 1× PBS. The “wounded” areas were photographed in specific wound sites (two sites for each wound) at 40× magnification using an Olympus IX51 inverted microscope equipped with an Olympus XM10 Digital Camera System every 24 h until wound closure. The relative migration distance (5 measures by wound) was calculated with the following formula: relative migration distance (%) = (A–B)/C × 100, where A is the width of cell wound at 0 h incubation, B is the width of cell wound after specific h of incubation, and C is the width mean of cell wound for 0 h of incubation. For relative migration distance, the results were analyzed using the beWound-Cell Migration Tool (Version 1.5) [50]. At least three independent experiments were performed.

### 4.15. Invasion Assay

Invasion capacity of sh-scramble/CTRL and sh-SIRT7 cells was evaluated using a 24 well BD Biocoat Matrigel Invasion Chambers (BD Biosciences, San Jose, CA, USA). After rehydration of BD Matrigel Chambers during 2 h with MEM medium at 37 °C, cells at a density of 25,000 cells/ insert were seeded and incubated during 24 h at 37 °C in 5% CO_2_. Then, the non-invading cells were removed by with swab and invaded cells were fixed with methanol and staining with DAPI. Invaded cells were counted on an Olympus IX51 fluorescence microscope equipped with an Olympus XM10 digital camera using CellSens software. The % invasion normalized for total of amount cell seeded in BD Matrigel Chamber.

### 4.16. Statistical Analysis

All statistical analyses were performed using IBM^®^ SPSS^®^ Statistic software version 23 (IBM-SPSS Inc., Chicago, IL, USA) and graphs were built using GraphPad Prim 7.0 (GraphPad Software Inc., La Jolla, CA, USA). Significance level was set at *p* < 0.05, and Bonferroni’s correction was used when appropriate. 

For both BlCa cohorts (IPO’s and TCGA), when applicable, Mann-Whitney U test (MW) was used to test for differences in sirtuins expression levels between NB and UCC tissue samples, pathological stages of cases divided in Ta-1 (NMIBC) and T2-4 (MIBC), and patients’ gender, and to assess differences in sh-scramble versus sh-SIRT7 conditions. Kruskall-Wallis test (KW) was performed to test for differences among UCC subtypes (papillary-low grade, papillary-high grade and invasive-high grade). Spearman’s rho was used to assess the correlation between SIRTs expression levels and age of the patients at diagnosis, and between SIRT7 and ECAD or NCAD expression levels. Associations between clinical grade or pathological stage and immunoexpression results were assessed by chi-square or Fisher’s exact test, and Somers’d directional measure was also computed. 

Disease-specific and disease-free survival curves (Kaplan-Meier with log rank test) were computed for standard variables (tumor stage and grade) and for categorized SIRTs transcript levels. Moreover, the same analyses were also performed separately for NMIBC and MIBC cases. A Cox-regression model comprising all significant variables (univariable and multivariable model) was computed to assess the relative contribution of each variable to the follow-up status.

## 5. Conclusions

In conclusion, this study provides a global view on sirtuin family expression deregulation in BlCa. Specifically, SIRT7 overexpression seems to play an important role in the first steps of urothelial carcinogenesis, whereas subsequent downregulation is associated with acquisition of an invasive and aggressive phenotype, through stimulation of EMT phenotype involving the SIRT7-EZH2-CDH1 axis. Although further studies are required to clarify the mechanism underlying SIRT7 deregulation in BlCa, it might constitute an attractive target for innovative therapeutic strategies.

## Figures and Tables

**Table 1:** Clinical and histopathological parameters of Bladder Cancer patients, and gender and age distribution of control set individuals.

Clinicopathological Features	Bladder UCC	Normal Bladder Mucosae
Patients, *n*	94	19
Gender, *n* (%)		
Males	78 (83)	19 (100)
Females	16 (17)	0
Median age, yrs (range)	69 (45–91)	63 (48–75)
Grade, *n* (%)		
Papillary, low-grade	33 (35)	n.a.
Papillary, high-grade	33 (35)	n.a.
Invasive, high-grade	28 (30)	n.a.
Pathological Stage, n (%)		
pTa/pT1 (NMIBC)	61 (65)	n.a.
pT2-4 (MIBC)	33 (35)	n.a.

(UCC—Urothelial Cell Carcinoma; yrs—years; NMIBC—non-muscle invasive bladder cancer; MIBC—muscle invasive bladder cancer).

**Table 2:** Clinical and histopathological parameters of bladder cancer patients, and gender and age distribution of control set individuals from TCGA cohort.

Clinicopathological Features	Bladder UCC	Matched Normal Bladder Mucosae
Patients, *n*	408	19
Gender, *n* (%)		
Males	301 (83)	10 (53)
Females	107 (17)	9 (47)
Median age, yrs (range)	69 (34–90)	71 (48–90)
Grade, *n* (%)		
Papillary, low-grade	18 (4)	n.a.
Papillary, high-grade	112 (28)	n.a.
Invasive, high-grade	278 (68)	n.a.
Pathological stage, *n* (%)		
pTa/pT1 (NMIBC)	2 (1)	n.a.
pT2-4 (MIBC)	406 (99)	n.a.

(UCC—Urothelial Cell Carcinoma; yrs—years; NMIBC—non-muscle invasive bladder cancer; MIBC—muscle invasive bladder cancer)

## Supplementary Materials

The following are available online at https://www.mdpi.com/2072-6694/12/5/1066/s1, Figure S1: Characterization of sirtuins’ family in the TCGA bladder cancer cohort; Figure S2: Characterization of Sirtuin family gene expression in the bladder cancer cohort and TCGA cohort categorized by clinical grade; Figure S3: Representative images for SIRT7 immunohistochemical expression in normal urothelium and bladder urothelial carcinoma tissue s tions; Figure S4: Representative images for SIRT7 immunofluorescent expression in MGHU3, 5637 and J82 bladder cancer cell lines; Figure S5: Representative images for E-Cadherin and N-Cadherin immunofluorescent expression in sh-CTRL and sh-SIRT7 MGHU3, 5637 and J82 bladder cancer cell lines; Figure S6: Characterization of *CDH1* and *CDH2* gene expression in bladder cancer and normal mucosae tissues, and in non-muscle invasive and muscle-invasive bladder cancer cases; Figure S7: Heat map showing *SIRT7* and *EZH2* expression in muscle invasive bladder cancer cases; Table S1: Reference of TaqMan^®^ gene expression assays for studied genes; Table S2: Primer sequences used in RT-PCR for studied genes, Figure S8: Detailed information about western blot Figure 3, Figure 4E and Figure 5C,E.
