# Supplementary material for: Sirtuins’ Deregulation in Bladder Cancer: SIRT7 Is Implicated in Tumor Progression through Epithelial to Mesenchymal Transition Promotion
Source: Cancers (Basel). 2020 Apr 25;12(5):1066. doi: 10.3390/cancers12051066 (PMC7281198; doi:10.3390/cancers12051066)
Supplement: Supplementary file 1 [file cancers-12-01066-s001.pdf]

Supplementary Materials

Sirtuins' Deregulation in Bladder Cancer: SIRT7 Is Implicated in Tumor Progression Through Epithelial to Mesenchymal Transition Promotion

Sara Monteiro-Reis, Ana Lameirinhas, Vera Miranda-Gonçalves, Diana Montezuma, Paula C. Dias, Jorge Oliveira, Inês Graça, Céline S. Gonçalves, Bruno M Costa, Rui Henrique and Carmen Jerónimo

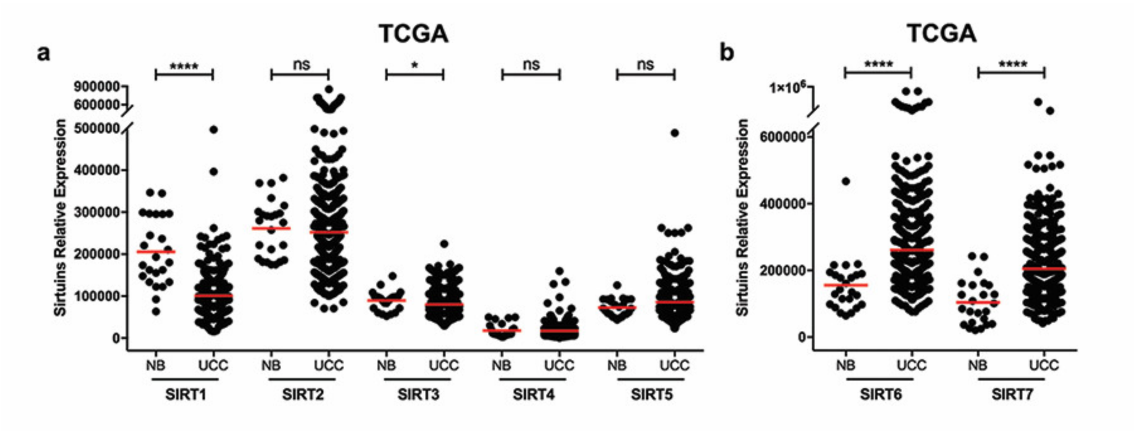

Figure S1: Characterization of SIRT1, SIRT2, SIRT3, SIRT4 and SIRT5 (**a**), and SIRT6 and SIRT7 (**b**) in the TCGA bladder cancer cohort by quantitative RT-PCR. * $p < 0.05$, **** $p < 0.0001$ and ns—non significant. UCC—urothelial cell carcinoma, NB—normal bladder mucosae.

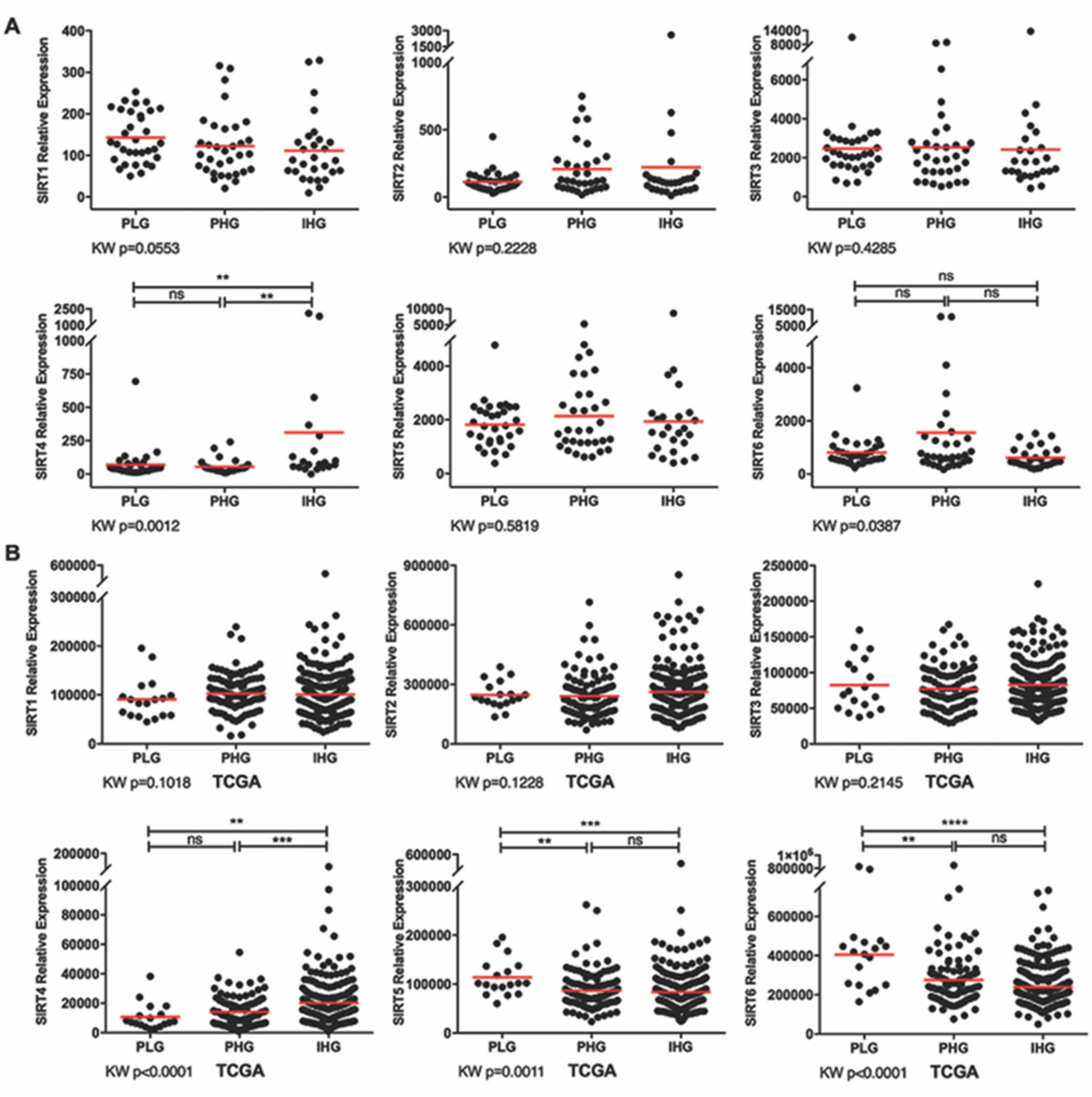

Figure S2: Characterization of Sirtuin family (SIRT1 to SIRT7) gene expression in bladder cancer tissues (**a**) and TCGA cohort (**b**) categorized by clinical grade. ** $p < 0.01$, *** $p < 0.001$, **** $p < 0.0001$ and ns – non significant. PLG – papillary low-grade, PHG – papillary high-grade, IHG – invasive high-grade.

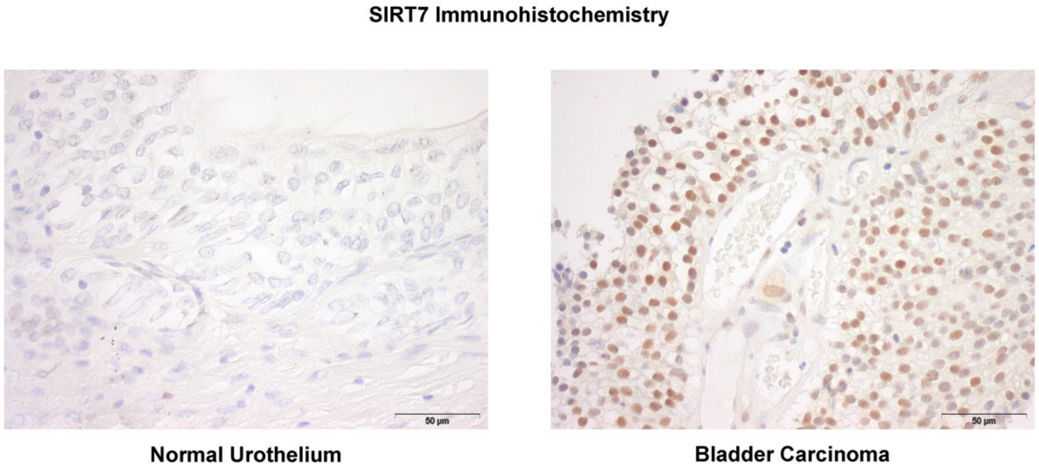

Figure S3: Immunohistochemistry representative images for SIRT7 immunoexpression in normal urothelium and bladder urothelial carcinoma tissue sections.

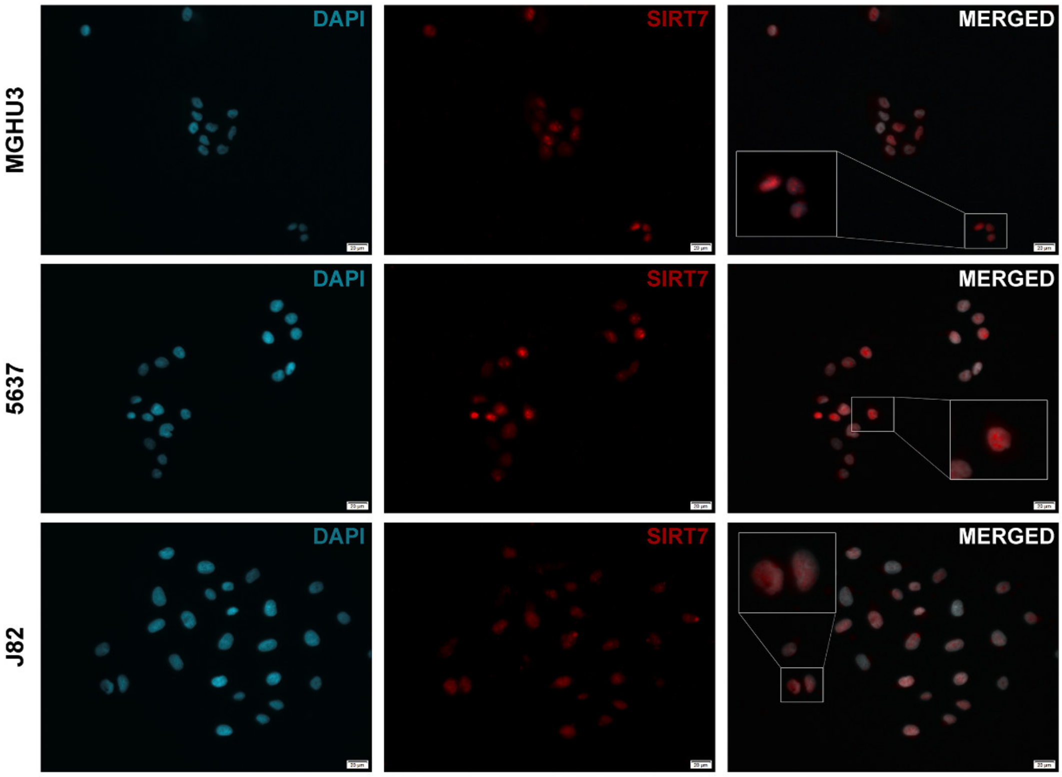

Figure S4. Immunofluorescence representative images for SIRT7 immunoexpression in MGHU3, 5637 and J82 bladder cancer cell lines (100× magnification).

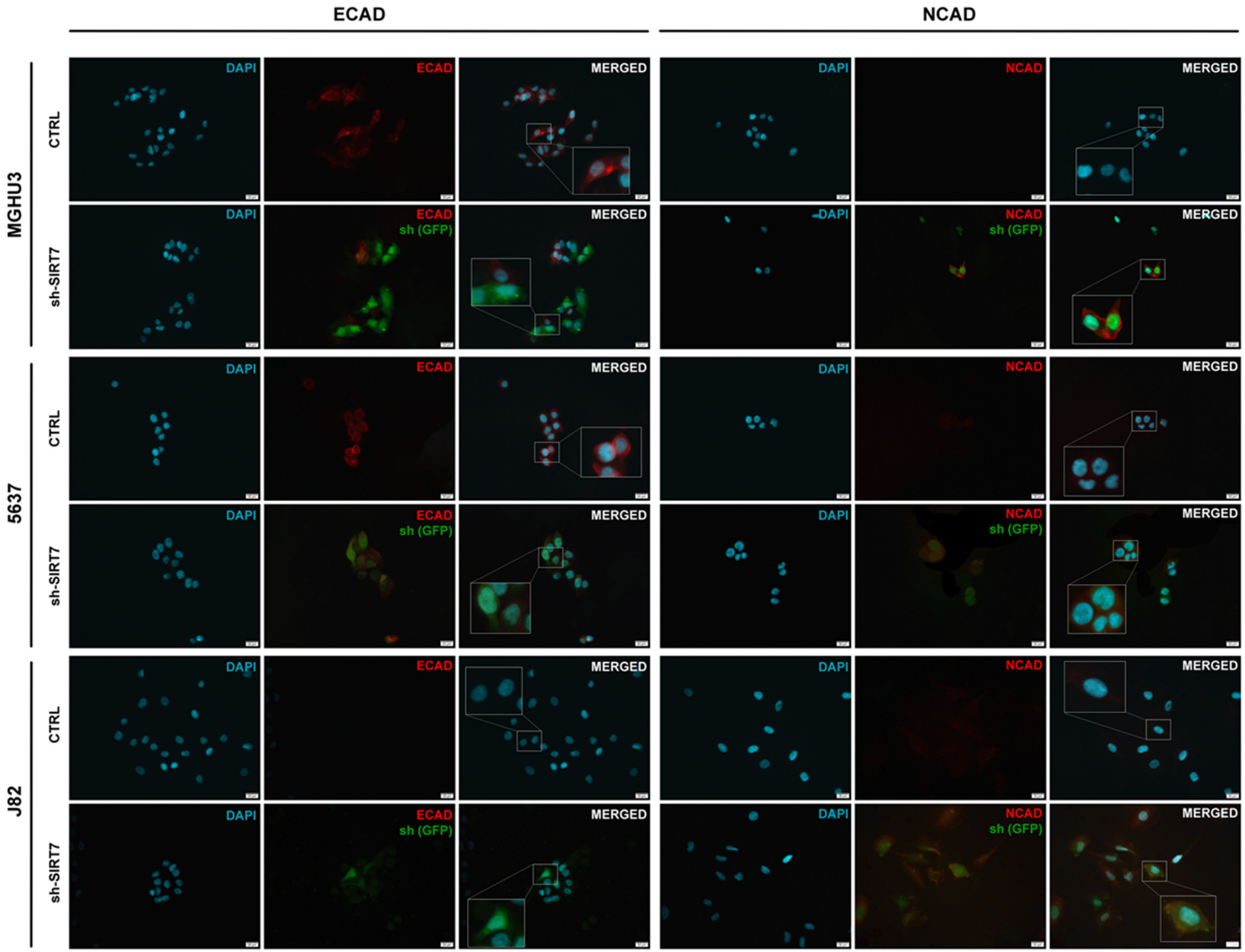

Figure S5: Immunofluorescence representative images for E-Cadherin and N-Cadherin immunoexpression in sh-CTRL and sh-SIRT7 MGHU3, 5637 and J82 bladder cancer cell lines (100× magnification).

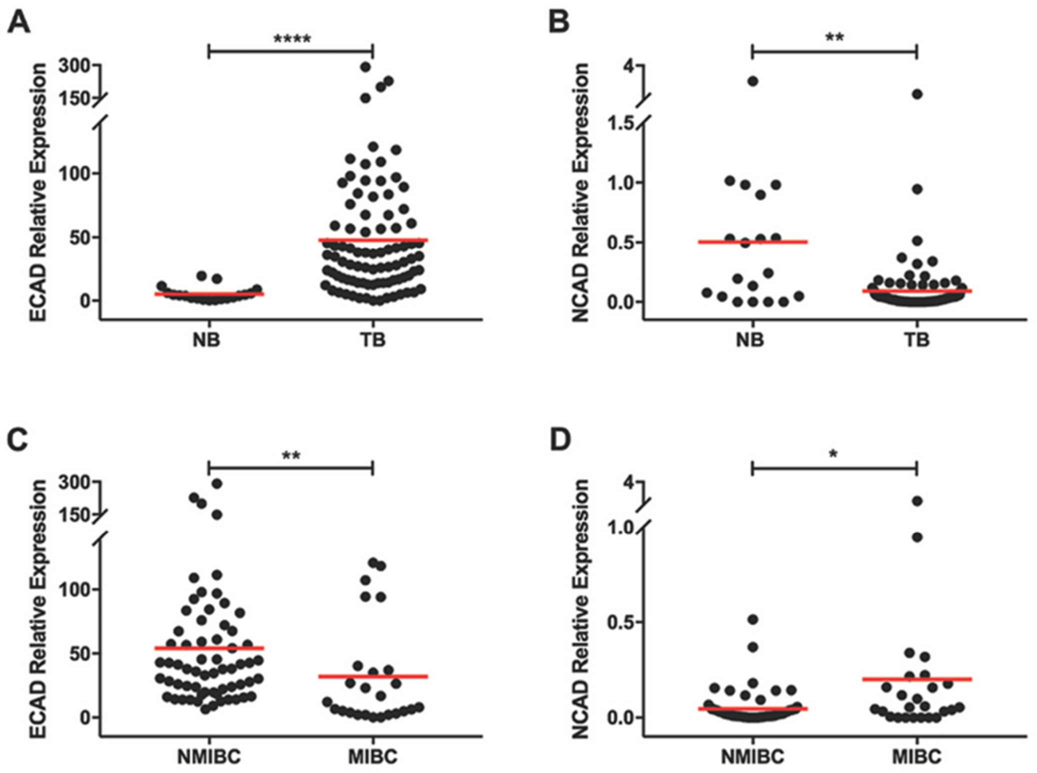

Figure S6. Characterization of *CDH1* gene expression in the bladder cancer and normal mucosae tissue samples (**a**), and in non-muscle invasive and muscle-invasive bladder cancer cases (**b**), by quantitative RT-PCR. Characterization of *CDH2* gene expression in the bladder cancer and normal mucosae tissue samples **(c)**, and in non-muscle invasive (NMIBC) and muscle-invasive bladder cancer (MIBC) cases **(d)**, by quantitative RT-PCR. * $p < 0.05$, ** $p < 0.01$ and **** $p < 0.0001$.

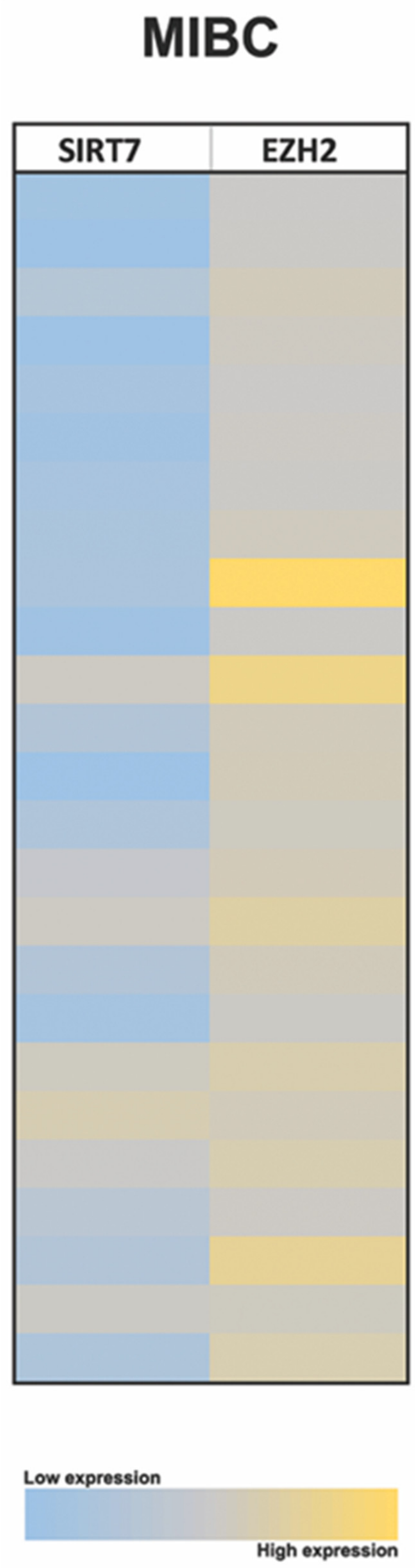

Figure S7. Heat map showing SIRT7 and EZH2 expression in muscle invasive bladder cancer (MIBC) cases (n = 26). Relative expression levels are depicted in colours from dark blue (lower expression), to dark yellow (higher expression) as shown in the colour bar bellow the graphic.

Table S1. Reference of TaqMan® gene expression assays for studied genes.

Gene	Reference ID
HPRT1	Hs01003267_m1
NCAD	Hs00354986_m1
SDHA	Hs00417200_m1
SIRT1	Hs01009005_m1
SIRT2	Hs00247263_m1
SIRT3	Hs00202030_m1
SIRT4	Hs00202033_m1
SIRT5	Hs00978335_m1
SIRT6	Hs00213036_m1
SIRT7	Hs01034735_m1

Table S2. Primer sequences used in RT-PCR for studied genes.

Gene	Forward sequence (5′-3′)	Reverse sequence (5′-3′)	Annealing T. (ºC)
BGUS	CTCATTTGGAATTTTGCCGATT	CCGAGTGAAGATCCCCTTTTTA	60
ECAD	CTTTGACGCCGAGAGCTACA	AAATTCACTCTGCCCAGGACG	64
EZH2	CCCTGACCTCTGTCTTACTTGTGGA	ACGTCAGATGGTGCCAGCAATA	60

		SIRT7 film					LMNB1 film					
Cell Line	Replicate	Band OD	Inverted	Background OD	Inverted	Net	Band OD	Inverted	Background OD	Inverted	Net	Ratio
SV-HUC1	1	193,40	61,60	200,98	54,02	7,58	179,91	75,09	212,41	42,59	32,500	0,233
	2	180,81	74,19	189,41	65,59	8,60	177,43	77,57	210,90	44,10	33,470	0,257
	3	194,35	60,65	201,28	53,72	6,93	181,27	73,73	212,05	42,95	30,780	0,225
MGHU3	1	170,27	84,73	201,77	53,23	31,50	177,91	77,09	212,92	42,08	35,010	0,900
	2	160,05	94,95	189,19	65,81	29,14	179,25	75,75	209,28	45,72	30,030	0,970
	3	168,89	86,11	202,08	52,92	33,19	181,14	73,86	212,94	42,06	31,800	1,044
5637	1	152,47	102,53	200,39	54,61	47,92	180,11	74,89	213,01	41,99	32,900	1,457
	2	150,90	104,10	190,42	64,58	39,52	178,41	76,59	209,79	45,21	31,380	1,259
	3	167,35	87,65	201,60	53,40	34,25	180,62	74,38	211,38	43,62	30,760	1,113
J82	1	171,12	83,88	199,98	55,02	28,86	179,32	75,68	212,69	42,31	33,370	0,865
	2	163,58	91,42	188,99	66,01	25,41	179,26	75,74	210,37	44,63	31,110	0,817
	3	174,10	80,90	201,04	53,96	26,94	182,98	72,02	212,59	42,41	29,610	0,910
T24	1	178,08	76,92	200,59	54,41	22,51	180,31	74,69	211,03	43,97	30,720	0,733
	2	168,92	86,08	189,08	65,92	20,16	177,39	77,61	209,82	45,18	32,430	0,622
	3	180,39	74,61	200,03	54,97	19,64	181,01	73,99	213,93	41,07	32,920	0,597
TCCSUP	1	201,13	53,87	201,37	53,63	0,24	179,38	75,62	212,46	42,54	33,080	0,007
	2	188,01	66,99	188,27	66,73	0,26	178,91	76,09	209,01	45,99	30,100	0,009
	3	202,71	52,29	202,86	52,14	0,15	179,95	75,05	211,93	43,07	31,980	0,005

			SIRT7 film					LMNB1 film								
	Cell Line	Replicate	Band OD	Inverted	Background OD	Inverted	Net	Band OD	Inverted	Background OD	Inverted	Net	Ratio	Mean Control	Fold Variation	Mean Fold
J82	sh-scramble	1	126,36	128,64	191,73	63,27	65,37	109,83	145,17	231,32	23,68	121,48	0,538	0,586	0,918	1,000
		2	131,03	123,97	203,28	51,72	72,25	115,07	139,94	231,05	23,95	115,99	0,623		1,063	
		3	127,45	127,55	199,12	55,88	71,67	110,89	144,11	230,91	24,09	120,02	0,597		1,019	
	sh-SIRT7	1	172,11	82,89	187,29	67,71	15,18	119,89	135,11	232,79	22,21	112,91	0,134		0,229	0,280
		2	175,27	79,73	204,53	50,47	29,26	106,00	149,00	230,33	24,67	124,33	0,235		0,402	
		3	177,35	77,65	191,79	63,21	14,44	109,42	145,58	228,19	26,81	118,77	0,122		0,207	
MGHU3	sh-CTRL	1	123,91	131,09	198,36	56,64	74,45	112,91	142,09	220,07	34,93	107,16	0,695	0,708	0,982	1,000
		2	127,10	127,90	200,72	54,28	73,62	115,02	139,98	218,34	36,66	103,32	0,713		1,007	
		3	124,56	130,44	195,27	59,73	70,71	123,13	131,87	221,90	33,10	98,77	0,716		1,012	
	sh-SIRT7	1	183,40	71,60	199,14	55,86	15,74	121,82	133,18	215,29	39,71	93,47	0,168		0,238	0,208
		2	181,09	73,91	194,25	60,75	13,16	118,29	136,71	222,39	32,61	104,10	0,126		0,179	
		3	181,55	73,45	194,78	60,22	13,23	120,04	134,96	221,06	33,94	101,02	0,131		0,185	
5637	sh-CTRL	1	136,38	118,62	197,58	57,42	61,20	134,23	120,77	227,62	27,39	93,38	0,655	0,603	1,087	1,000
		2	125,10	129,90	185,46	69,54	60,36	122,29	132,71	228,41	26,59	106,12	0,569		0,944	
		3	131,93	123,07	189,21	65,79	57,28	131,72	123,28	229,81	25,19	98,09	0,584		0,969	
	sh-SIRT7	1	171,10	83,91	190,73	64,27	19,64	131,46	123,54	229,19	25,81	97,73	0,201		0,333	0,458
		2	162,42	92,58	187,15	67,85	24,73	127,40	127,60	225,27	29,73	97,87	0,253		0,419	
		3	155,81	99,19	191,92	63,08	36,11	130,62	124,38	227,01	27,99	96,39	0,375		0,622	

MGHU3		ECAD film					Actin film					Ratios		
		Band OD	inverted	background OD	inverted	Net	Band OD	inverted	background OD	inverted	Net	ECAD/ACTIN		
sh-CTRL	(1)	216,70	38,30	233,91	21,09	17,21	159,28	95,72	170,91	84,09	11,63	1,480	2,976	0,469
	(2)	117,20	137,80	191,99	63,01	74,80	161,42	93,58	179,95	75,05	18,53	4,036		1,279
	(3)	193,91	61,09	226,72	28,28	32,82	159,93	95,07	169,54	85,46	9,61	3,413		1,082
sh-SIRT7	(1)	221,92	33,08	251,34	3,66	29,42	155,83	99,17	171,40	83,60	15,57	1,890		0,599
	(2)	203,85	51,15	229,95	25,05	26,10	157,73	97,27	171,08	83,92	13,36	1,954		0,619
	(3)	207,68	47,32	240,11	14,89	32,43	154,34	100,66	170,41	84,59	16,07	2,018		0,639

MGHU3		NCAD film					Actin film					Ratios		
		Band OD	inverted	background OD	inverted	Net	Band OD	inverted	background OD	inverted	Net	NCAD/ACTIN		
sh-CTRL	(1)	195,12	59,88	238,63	16,37	43,51	159,28	95,72	170,91	84,09	11,63	3,742	3,156	1,257
	(2)	168,54	86,46	223,185	31,82	54,65	161,42	93,58	179,95	75,05	18,53	2,949		0,991
	(3)	185,14	69,86	211,837	43,16	26,69	159,93	95,07	169,54	85,46	9,61	2,777		0,933
sh-SIRT7	(1)	165,81	89,19	253,91	1,09	88,10	155,83	99,17	171,40	83,60	15,57	5,658		1,793
	(2)	170,73	84,27	245,33	9,67	74,60	157,73	97,27	171,08	83,92	13,36	5,586		1,770
	(3)	153,56	101,44	240,11	14,89	86,55	154,34	100,66	170,41	84,59	16,07	5,385		1,706

MGHU3		SLUG film					Actin film					Ratios		
		Band OD	inverted	background OD	inverted	Net	Band OD	inverted	background OD	inverted	Net	SLUG/ACTIN		
sh-CTRL	(1)	193,82	61,18	194,76	60,24	0,94	159,28	95,72	170,91	84,09	11,63	0,081	1,991	0,041
	(2)	133,47	121,53	202,54	52,46	69,07	161,42	93,58	179,95	75,05	18,53	3,727		1,872
	(3)	179,64	75,36	200,46	54,54	20,81	159,93	95,07	169,54	85,46	9,61	2,165		1,087
sh-SIRT7	(1)	129,70	125,30	185,24	69,76	55,55	155,83	99,17	171,40	83,60	15,57	3,568		1,792
	(2)	134,51	120,49	183,11	71,89	48,61	157,73	97,27	171,08	83,92	13,36	3,639		1,828
	(3)	187,27	67,73	246,23	8,77	58,96	154,34	100,66	170,41	84,59	16,07	3,668		1,842

MGHU3		SNAIL film					Actin film					Ratios		
		Band OD	inverted	background OD	inverted	Net	Band OD	inverted	background OD	inverted	Net	SNAIL/ACTIN		
sh-CTRL	(1)	208,21	46,79	252,52	2,48	44,31	159,28	95,72	170,91	84,09	11,63	3,811	5,921	0,644
	(2)	134,76	120,24	253,67	1,33	118,91	161,42	93,58	179,95	75,05	18,53	6,417		1,084
	(3)	178,10	76,90	250,54	4,46	72,44	159,93	95,07	169,54	85,46	9,61	7,535		1,273
sh-SIRT7	(1)	148,16	106,84	250,74	4,26	102,58	155,83	99,17	171,40	83,60	15,57	6,589		1,113
	(2)	158,70	96,30	252,91	2,09	94,21	157,73	97,27	171,08	83,92	13,36	7,054		1,191
	(3)	139,49	115,51	253,85	1,15	114,36	154,34	100,66	170,41	84,59	16,07	7,115		1,202

5637		ECAD film					Actin film					Ratios		
		Band OD	inverted	background OD	inverted	Net	Band OD	inverted	background OD	inverted	Net	ECAD/ACTIN		
sh-CTRL	(1)	180,89	74,11	230,77	24,23	49,88	175,31	79,69	191,33	63,67	16,02	3,114	2,802	1,111
	(2)	154,13	100,88	227,56	27,44	73,44	173,95	81,05	192,47	62,53	18,52	3,965		1,415
	(3)	212,40	42,60	230,62	24,38	18,22	177,56	77,44	191,28	63,72	13,72	1,328		0,474
sh-SIRT7	(1)	206,51	48,49	231,23	23,77	24,72	172,34	82,66	190,21	64,79	17,87	1,383		0,494
	(2)	202,67	52,33	233,71	21,29	31,04	169,27	85,73	191,08	63,92	21,81	1,423		0,508
	(3)	205,21	49,79	230,89	24,11	25,68	173,88	81,12	192,90	62,10	19,02	1,350		0,482

5637		NCAD film					Actin film					Ratios		
		Band OD	inverted	background OD	inverted	Net	Band OD	inverted	background OD	inverted	Net	NCAD/ACTIN		
sh-CTRL	(1)	143,79	111,21	191,34	63,66	47,55	175,31	79,69	191,33	63,67	16,02	2,968	2,801	1,059
	(2)	150,44	104,56	193,89	61,11	43,45	173,95	81,05	192,47	62,53	18,52	2,346		0,837
	(3)	149,27	105,73	191,66	63,34	42,39	177,56	77,44	191,28	63,72	13,72	3,090		1,103
sh-SIRT7	(1)	113,83	141,17	191,21	63,79	77,38	172,34	82,66	190,21	64,79	17,87	4,330		1,546
	(2)	102,74	152,26	192,99	62,01	90,25	169,27	85,73	191,08	63,92	21,81	4,138		1,477
	(3)	114,01	140,99	192,17	62,83	78,16	173,88	81,12	192,90	62,10	19,02	4,109		1,467

5637		SLUG film					Actin film					Ratios		
		Band OD	inverted	background OD	inverted	Net	Band OD	inverted	background OD	inverted	Net	SLUG/ACTIN		
sh-CTRL	(1)	157,99	97,01	200,44	54,56	42,45	175,31	79,69	191,33	63,67	16,02	2,650	2,691	0,985
	(2)	162,41	92,59	205,79	49,21	43,38	173,95	81,05	192,47	62,53	18,52	2,342		0,871
	(3)	160,87	94,13	203,12	51,88	42,25	177,56	77,44	191,28	63,72	13,72	3,079		1,145
sh-SIRT7	(1)	125,71	129,29	203,57	51,43	77,86	172,34	82,66	190,21	64,79	17,87	4,357		1,619
	(2)	107,22	147,78	200,91	54,09	93,69	169,27	85,73	191,08	63,92	21,81	4,296		1,597
	(3)	116,58	138,42	200,32	54,68	83,74	173,88	81,12	192,90	62,10	19,02	4,403		1,636

5637		SNAIL film					Actin film					Ratios		
		Band OD	inverted	background OD	inverted	Net	Band OD	inverted	background OD	inverted	Net	SNAIL/ACTIN		
sh-CTRL	(1)	169,59	85,41	245,93	9,07	76,34	175,31	79,69	191,33	63,67	16,02	4,765	4,873	0,978
	(2)	161,33	93,67	243,60	11,40	82,27	173,95	81,05	192,47	62,53	18,52	4,442		0,912
	(3)	171,20	83,80	245,44	9,56	74,24	177,56	77,44	191,28	63,72	13,72	5,411		1,110
sh-SIRT7	(1)	89,90	165,10	243,71	11,29	153,81	172,34	82,66	190,21	64,79	17,87	8,607		1,766
	(2)	60,61	194,39	245,99	9,01	185,38	169,27	85,73	191,08	63,92	21,81	8,500		1,744
	(3)	79,55	175,45	241,87	13,13	162,32	173,88	81,12	192,90	62,10	19,02	8,534		1,751

J82		NCAD film					Actin film					Ratios		
		Band OD	inverted	background OD	inverted	Net	Band OD	inverted	background OD	inverted	Net	NCAD/ACTIN		
sh-scramble	(1)	127,90	127,10	188,04	66,96	60,14	162,23	92,77	182,76	72,24	20,53	2,929	2,515	1,165
	(2)	134,65	120,35	190,21	64,79	55,56	155,12	99,88	183,94	71,06	28,82	1,928		0,767
	(3)	131,19	123,81	189,14	65,86	57,95	160,44	94,56	182,01	72,99	21,57	2,687		1,068
sh-SIRT7	(1)	104,51	150,49	188,37	66,63	83,86	161,91	93,09	180,39	74,61	18,48	4,538		1,805
	(2)	81,12	173,88	186,29	68,71	105,17	159,30	95,70	182,22	72,78	22,92	4,589		1,825
	(3)	91,72	163,28	189,01	65,99	97,29	161,19	93,81	182,17	72,83	20,98	4,637		1,844

J82		SNAIL film					Actin film					Ratios		
		Band OD	inverted	background OD	inverted	Net	Band OD	inverted	background OD	inverted	Net	SNAIL/ACTIN		
sh-scramble	(1)	169,59	85,41	240,37	14,63	70,78	162,23	92,77	182,76	72,24	20,53	3,448	3,151	1,094
	(2)	161,33	93,67	241,24	13,76	79,91	155,12	99,88	183,94	71,06	28,82	2,773		0,880
	(3)	171,20	83,80	240,90	14,10	69,70	160,44	94,56	182,01	72,99	21,57	3,231		1,026
sh-SIRT7	(1)	153,10	101,90	238,73	16,27	85,63	161,91	93,09	180,39	74,61	18,48	4,634		1,471
	(2)	133,41	121,59	240,99	14,01	107,58	159,30	95,70	182,22	72,78	22,92	4,694		1,490
	(3)	145,02	109,98	241,04	13,96	96,02	161,19	93,81	182,17	72,83	20,98	4,577		1,453

5637		EZH2 film					LMNB1 film					Ratios		
		Band OD	inverted	Background OD	inverted	Net	Band OD	inverted	Background OD	inverted	Net	EZH2/LMNB1		
sh-CTRL	(1)	180,89	74,11	222,99	32,01	42,10	182,30	72,70	219,01	35,99	36,71	1,147	0,989	1,160
	(2)	154,13	100,88	219,94	35,06	65,82	181,34	73,66	220,82	34,18	39,48	1,667		1,686
	(3)	212,40	42,60	217,38	37,62	4,98	184,88	70,12	217,53	37,47	32,65	0,153		0,154
sh-SIRT7	(1)	169,47	85,53	241,38	13,62	71,91	180,39	74,61	221,28	33,72	40,89	1,759		1,779
	(2)	176,30	78,70	239,28	15,72	62,98	182,91	72,09	219,06	35,94	36,15	1,742		1,762
	(3)	178,17	76,83	240,25	14,75	62,08	182,49	72,51	218,48	36,52	35,99	1,725		1,744

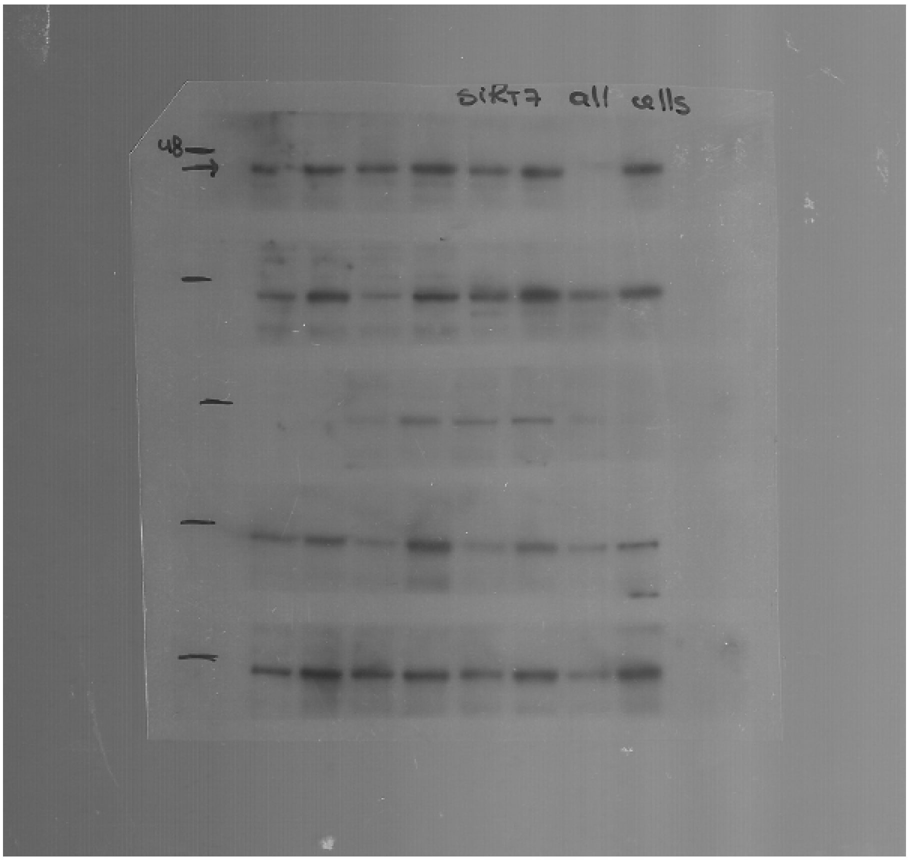
SIRT7 all cells
48

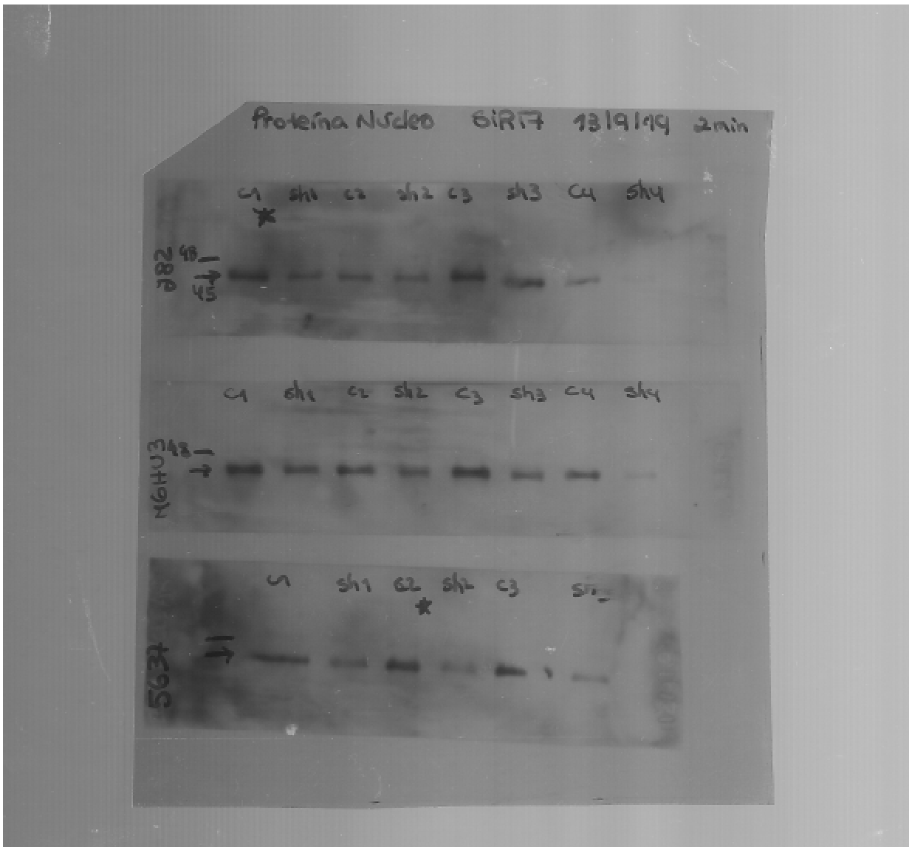
Proteina Nucleo SIRT7 13/9/19 2min
c1 sh1 c2 sh2 c3 sh3 c4 sh4
J82
48
45
c1 sh1 c2 sh2 c3 sh3 c4 sh4
MGHU3
48
c1 sh1 c2 sh2 c3
5637

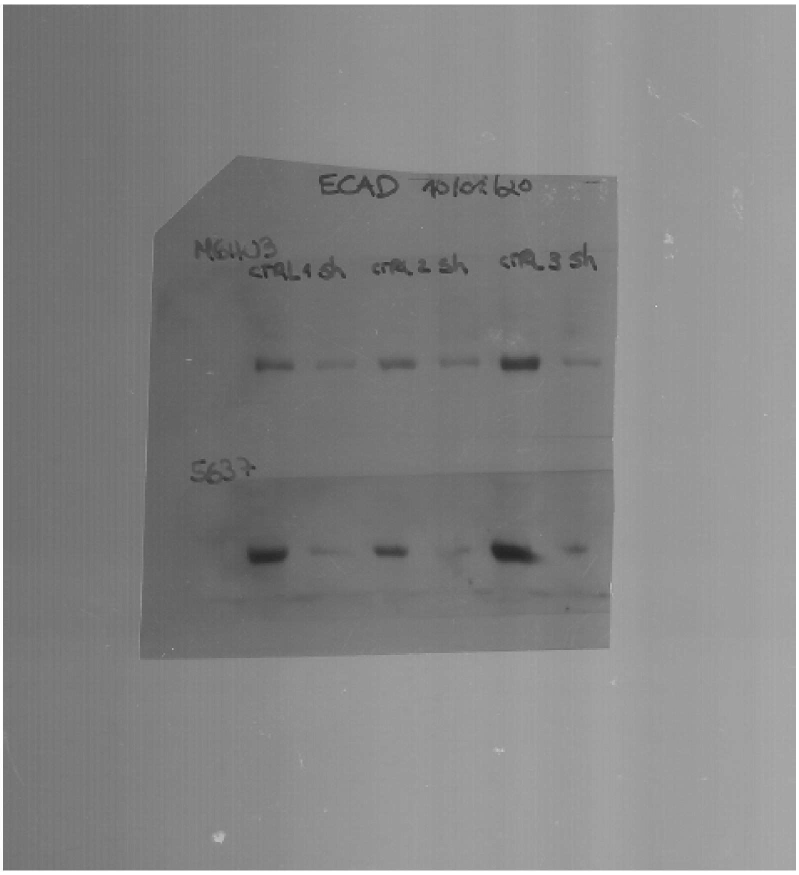
ECAD 10/01/20
MGHU3
ctRL 1 sh ctRL 2 sh ctRL 3 sh
5637

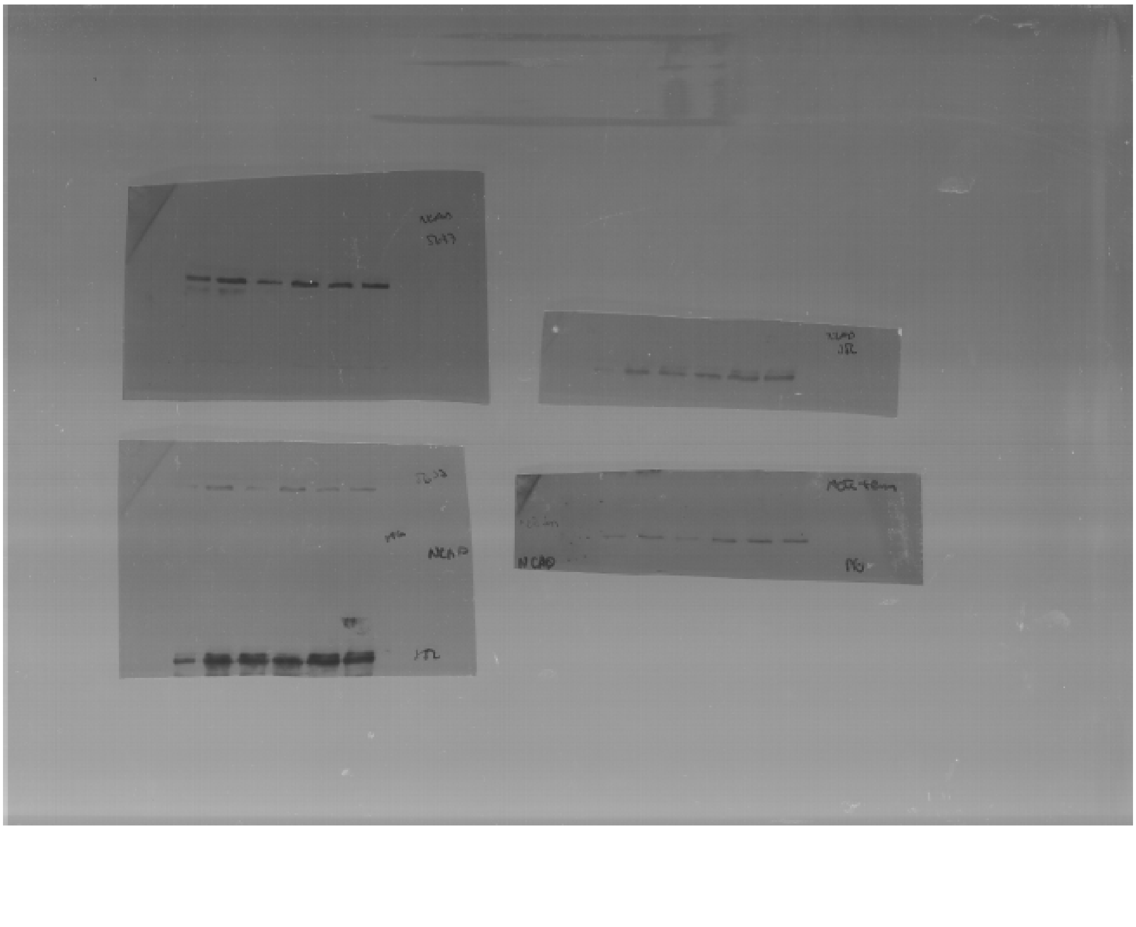

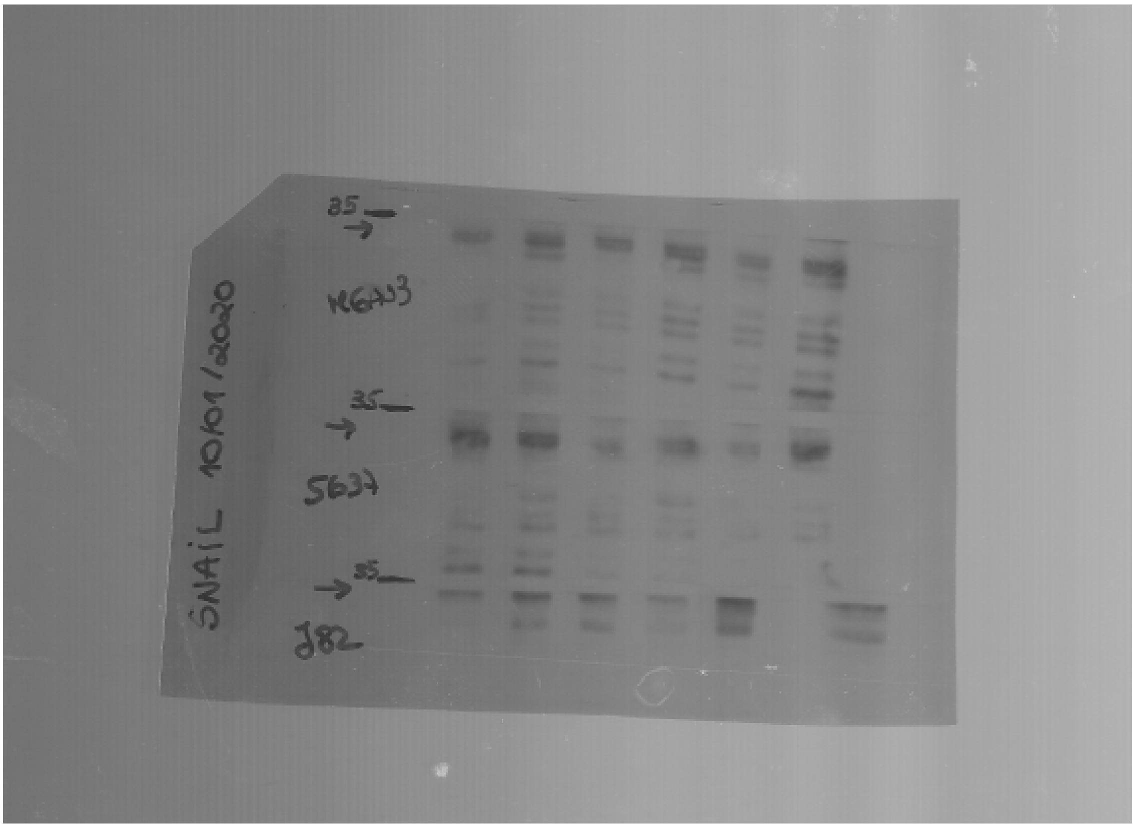
SNAIL 10/01/2020
35
5637
35
35
J82

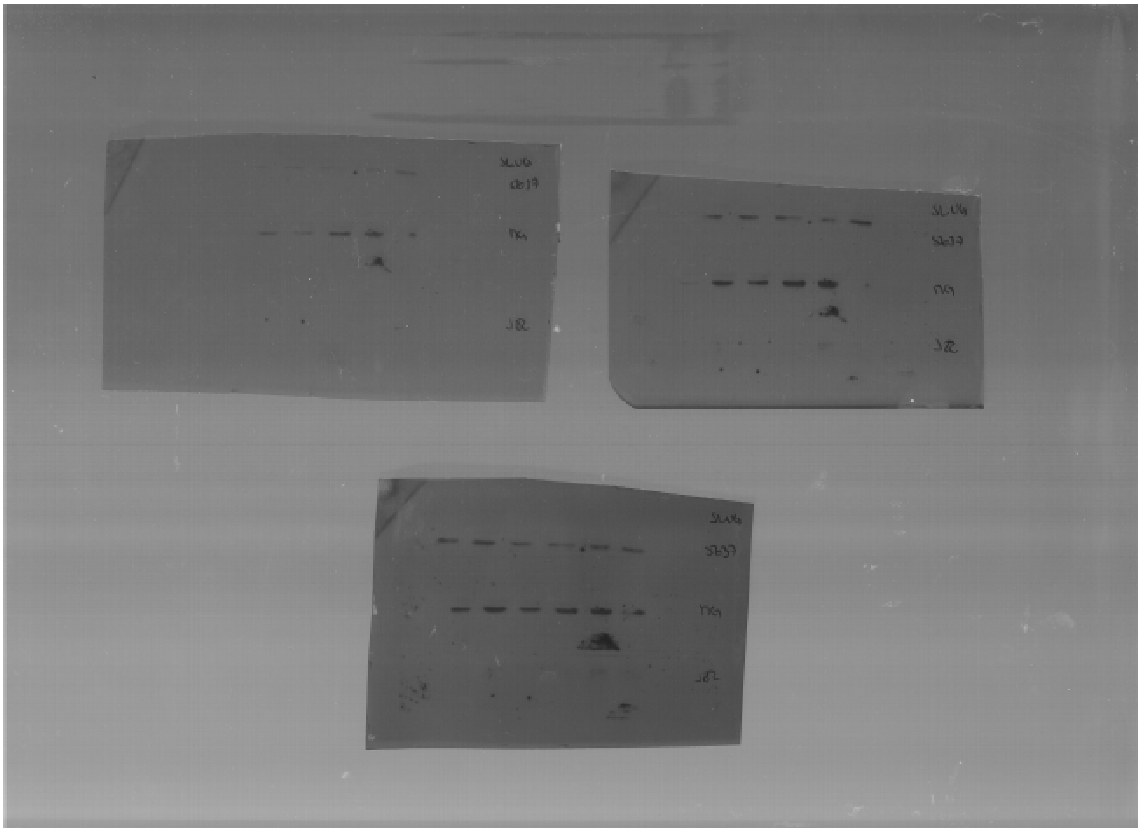
SLUG
5637
J82

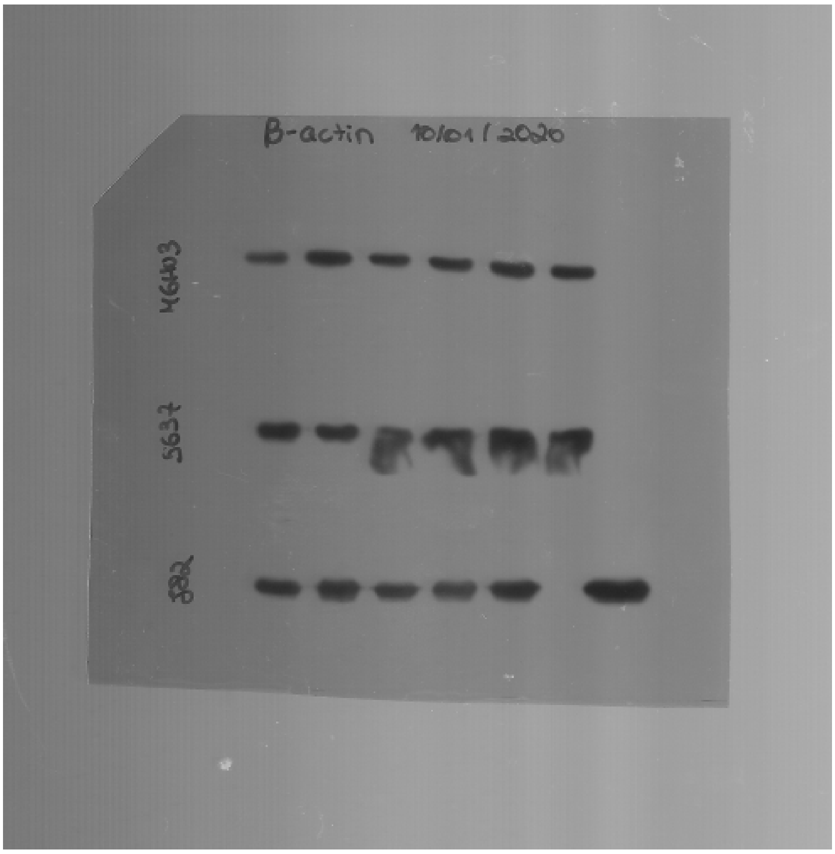
β-actin 10/01/2020
MGHU3
5637
J82

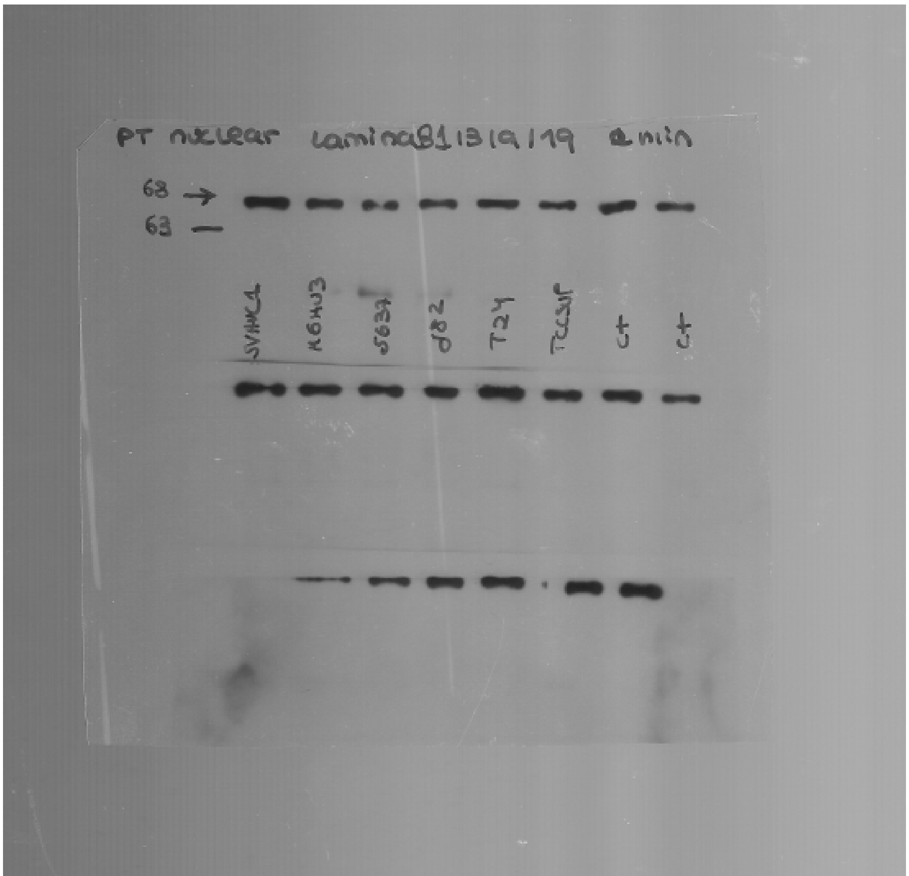
PT nuclear laminaB1 13/9/19
68
63
SVHUC1
MGHU3
5637
J82
T24
TCCSUP
c+
c+

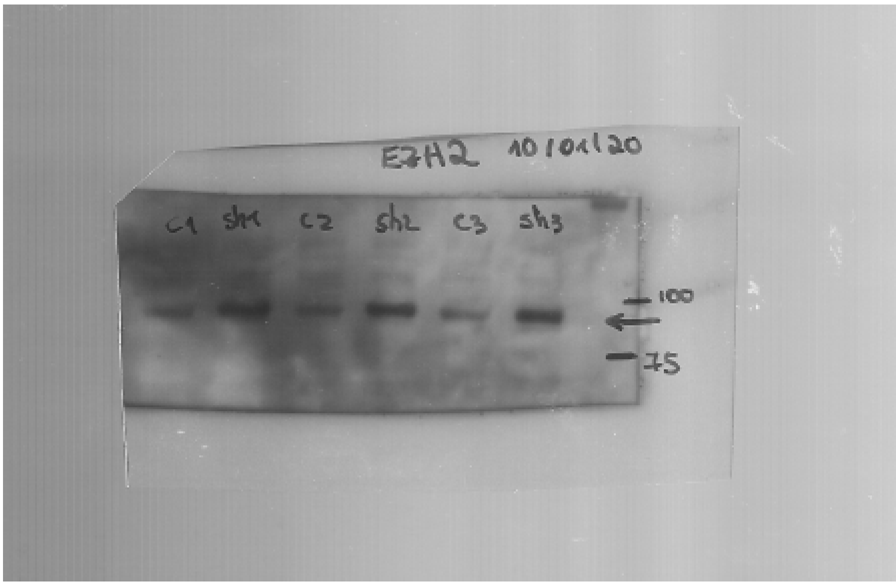

Figure S8. Detailed Information about Western Blot Figures 3, 4E and 5F.

© 2020 by the authors. Licensee MDPI, Basel, Switzerland. This article is an open access article distributed under the terms and conditions of the Creative Commons Attribution (CC BY) license (http://creativecommons.org/licenses/by/4.0/).
